# Deep belief rule based photovoltaic power forecasting method with interpretability

**DOI:** 10.1038/s41598-022-18820-6

**Published:** 2022-08-24

**Authors:** Peng Han, Wei He, You Cao, YingMei Li, YunYi Zhang

**Affiliations:** 1 Harbin Normal University, Harbin, 150025 China; 2 Rocket Force University of Engineering, Xi’an, 710025 China

**Keywords:** Engineering, Mathematics and computing

## Abstract

Accurate prediction of photovoltaic (PV) output power is of great significance for reasonable scheduling and development management of power grids. In PV power generation prediction system, there are two problems: the uncertainty of PV power generation and the inexplicability of the prediction result. The belief rule base (BRB) is a rule-based modeling method and can deal with uncertain information. Moreover, the modeling process of BRB has a certain degree of interpretability. However, rule explosion and the inexplicability of the optimized model limit the modeling ability of BRB in complex systems. Thus, a PV output power prediction model is proposed based on a deep belief rule base with interpretability (DBRB-I). In the DBRB-I model, the deep BRB structure is constructed to solve the rule explosion problem, and inefficient rules are simplified by a sensitivity analysis of the rules, which reduces the complexity of the model. Moreover, to ensure that the interpretability of the model is not destroyed, a new optimization method based on the projection covariance matrix adaptation evolution strategy (P-CMA-ES) algorithm is designed. Finally, a case study of the prediction of PV output power is conducted to illustrate the effectiveness of the proposed method.

## Introduction

Photovoltaic (PV) power generation has developed rapidly due to its clean energy characteristics. However, PV power output is affected by external uncertain factors, such as solar irradiance, voltage, module temperature, and ambient temperature. Its power output has fluctuation, randomness and uncertainty^1^. These characteristics will cause many problems for the safe operation and reasonable dispatch of the power grid^2^. Therefore, an accurate PV power forecasting system is crucial for the grid dispatch center to make decisions such as storage requirements, scheduling arrangements, and balancing supply and demand in the power market^3^.

In current research, three main methods can be found in PV power prediction: physical models, statistical models and hybrid approaches^4^. The physical model is used to calculate the main design parameters of the PV power generation system. Chen et al. proposed a spatio-temporal (ST) PV power nowcasting method with predictor preselection; this method enables fast and accurate PV nowcasts in different scenarios^5^. Wen et al. proposed a novel multistep forecasting (MSF) scheme, which can make multiple predictions while maintaining the same high temporal resolution^6^. Moreover, Saint-Drenan et al. proposed an empirical method for the parameterization of PV power plants for power prediction; this model makes full use of the information commonly available in PV plants^7^. Almeida et al. used a mathematical model with multiple parameters to predict PV power generation, but prediction accuracy relies on multiple mathematical models capable of describing PV system parameters^8^. The accuracy of the PV power prediction physical model relies on accurate meteorological data and complete PV battery information. However, due to the incomplete parameters provided by manufacturers and the limited prediction accuracy of numerical weather prediction (NWP), the modeling accuracy based on physical models is limited^4,9^.

The statistical model is established by learning the law of observational data. For exapmple, Miao et al. proposed a Markov chain model to evaluate solar power generation performance; this method fully investigates the effects of seasonal solar profile patterns and PV module types on solar power generation^10^. Massidda et al. used multilinear adaptive regression splines and NWP to predict PV power output, and the model has regression coefficients that are easy to interpret^11^. Moreover, Rodriguez et al. proposed an artificial neural network (ANN) to predict the amount of solar energy generated by PV generators, which effectively solves the complexity of control in solar energy systems^12^. Yagli et al. used 68 machine learning models to automate hourly sun forecasts, and the comparative analysis proves that the tree-based method performs well in terms of overall results^13^. However, statistical models based on data-driven methods are black-box models whose output results are not interpretable. The hybrid method refers to the combination of two different methods. Halabi et al. proposed a hybrid adaptive neuro-fuzzy inference system model for the efficient prediction of solar radiation, and the hybrid model can accurately predict solar radiation according to different meteorological parameters, such as sunshine hours and temperature^14^. Barman et al. proposed a season-specific method for short-term load forecasting based on hybrid firefly algorithm-support vector machine (FA-SVM); the method takes into account seasonal effects and has excellent predictive power in all cases^15^. Hybrid physics and data-driven modeling approaches provide high accuracy in forecasting PV power generation forecasting techniques^4^. Moreover, most of the research focuses on point prediction^1,5,9^, the result of point prediction is a specific value at a certain prediction moment, and the prediction result obtained by point prediction is intuitive compared with probability prediction. Intuitive prediction results can help grid operators make faster decisions about grid dispatch.

In PV power prediction system, two problems need to be solved to construct an accurate and reliable prediction system. First, PV power generation is affected by many meteorological factors, such as solar irradiance, ambient temperature, and module temperature^16,17^. Due to the inherent uncertainty of meteorology, PV power generation is uncertain, fluctuating and intermittent^2^. These stochastic behaviors create many problems for the power dispatching and security of the power grid^3^. Thus, the constructed PV power prediction system needs to have the ability to deal with uncertain information. Second, PV power generation is the main energy source of the power grid. The main requirements for grid management are reliability and efficiency, and grid operators need fast and accurate knowledge of the power supply^18^. Therefore, PV power output needs to be forecasted in an interpretable way, which can help grid operators make reasonable decisions and make operators trust the model^19^. BRB can effectively solve the above problems, and BRB has nonlinear modelling ability, which can effectively deal with the coexistence of uncertain information. BRB based on the IF–THEN rule modelling method can describe the modelling process and decision-making process in linguistic terms^20^. Moreover, BRB has a transparent reasoning process based on the evidence reasoning (ER) algorithm, and grid operators can directly touch and access the model^21^.

However, there are three problems in the PV power output prediction system based on BRB. First, PV power generation is affected by many attributes, but the number of BRB rules is generated as the Cartesian product of the attribute reference values, and the number of BRB rules increases exponentially, which causes the problem of rule explosion. Moreover, in a PV power generation system based on BRB, the input attributes vary with seasons and geographic locations. Thus, expert knowledge needs to be reluctantly redesigned as new attribute information is added to the model^22^. Second, the initial BRB model constructed by experts is subjective, so the BRB model needs to be optimized through observational data. However, the interpretability of the BRB model will be destroyed due to the randomness of the algorithm^23^. Third, there are many inefficient rules in the BRB's rule base, which reduces the readability and interpretability of the BRB model. Thus, it is important to implement rule reduction on the rule base in a reliable way^23^. Hence, a new prediction model deep belief rule base with interpretability (DBRB-I) is proposed to solve the above problems. The DBRB-I model is composed of multiple Sub-BRBs in a deep structure, which effectively solves the rule explosion problem and weak extensibility. The DBRB-I model uses a new optimization method based on the projection covariance matrix adaptation evolution strategy (P-CMA-ES) algorithm to ensure the interpretability of the model after optimization. Moreover, the DBRB-I model implements rule reduction in a reliable manner by performing a sensitivity analysis on the initial BRB model.

The main contributions of this paper include the following: (1) The DBRB-I model is proposed to predict photovoltaic power output in an interpretable manner. (2) A new optimization algorithm with interpretability is designed. (3) A sensitivity analysis method is proposed to implement rule reduction.

The remainder of this paper is organized as follows. In “Problem description” section, the problems of PV power generation systems are formulated, and a new prediction model based on DBRB-I is proposed. The interpretability of the DBRB-I model is introduced in “The DBRB-I model interpretability” section, including interpretability criteria and interpretability constraints. In “Sensitivity analysis” section, sensitivity analysis methods are introduced. The reasoning process and optimization process of the DBRB-I model are given in “Reasoning process and optimization process of the DBRB-I model” section. Then, a case study is conducted to verify the effectiveness of the proposed model in “Case study” section. This paper is concluded in “Conclusion” section.

## Problem description

Before starting the work in this paper, a hypothesis needs to be formulated. The initial modeling assumption for the interpretability model is that expert knowledge is reliable. Expert knowledge is obtained through the analysis of the mechanism operation of PV power generation systems and the accumulation of long-term knowledge. Although expert knowledge has certain limitations in accuracy, the direction of expert knowledge must be correct. Therefore, the initial model of this paper is constructed with reliable and reasonable expert knowledge.

In “Problems with prediction systems” section, the problem of predicting PV output power is formulated. Then, in “Construction of the DBRB-I model” section, a prediction model of PV output power based on DBRB-I was constructed.

### Problems with prediction systems

To build a PV output power prediction model based on DBRB-I, the following three problems need to be solved*.*

#### Problem 1

Build a new extensible BRB model. Expert knowledge is crucial in the BRB expert system, which can effectively ensure the interpretability and accuracy of the model. However, due to the complexity of PV power generation systems, the number of rules increases exponentially with the number of attribute referential values. This has led to the problem of rule explosion, which reduces the interpretability and readability of the model. Meanwhile, input attributes vary with season and geographic location in PV power generation system, and weak extendability can cause expert knowledge to be reluctant to be redesigned when new attribute information is used. Therefore, the first question is how to build a new BRB model, which has good extendability and can avoid the combination explosion problem.

1 $$y = f(x,C,\Omega ,Ek)$$

where $x$ is the input data of the PV power generation system,$C$ is the set of interpretability constraints,$\Omega$ is the model parameter,$Ek$ is the expert knowledge introduced into the model, and $y$ is the predicted result of PV power generation systems.

#### Problem 2

Interpretability constraints are designed. BRB is a rule-based modelling method that can easily understand the modelling process of the model. However, the interpretability of the model optimization process will be destroyed due to the randomness of the optimization algorithm. Thus, the second problem is how to design effective interpretability constraints to ensure that the interpretability of the model is not destroyed.

2 $$Interpretability:\left\{ {C|C_{1} ,C_{2} ,...C_{m} } \right\}$$

3 $$\Omega = optimize(x,y,P,C)$$

where $P$ is the set of parameters in the optimization process.

#### Problem 3

Improve the simplicity of the rules base. The rule base in BRB is generated by the attribute reference value in the form of Descartes. Some redundant rules and inefficient rules exist in the rules base of BRB, which will reduce the accuracy and readability of the model. Therefore, the third question is how to reasonably and transparently remove redundant rules and inefficient rules in the BRB rule base.

4 $$g = \left| {MSE_{SA} - MSE_{{({\text{initial}})}} } \right|$$

where $g$ is the error fluctuation of the model,$MSE_{SA}$ is the accuracy of the model after sensitivity analysis, and $MSE_{{({\text{initial}})}}$ is the accuracy of the model built with expert knowledge.

### Construction of the DBRB-I model

**Figure 1:**
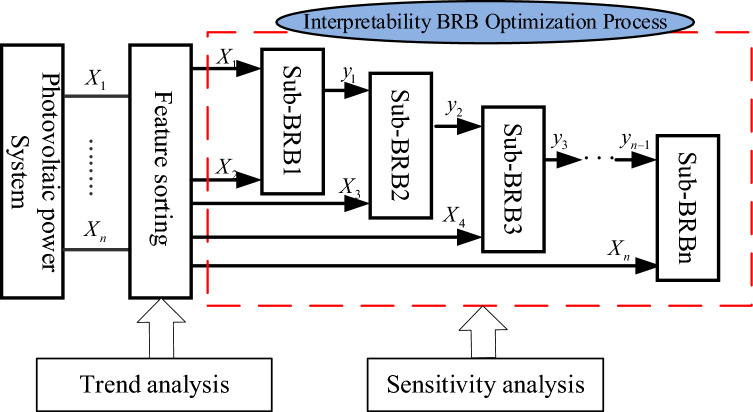
The DBRB-I model based on the PV power generation system.

BRB is composed of a series of belief rules, and the kth rule can be described as follows:

5 $$\begin{gathered} IF \, x_{1} {\text{ is A}}_{1}^{k} \wedge x_{2} {\text{ is A}}_{2}^{k} \wedge \cdots \wedge x_{{T_{k} }} {\text{ is A}}_{{T_{k} }}^{k} , \, \hfill \\ {\text{THEN }}y \, is\{(D_{1} {,}\beta_{1,k} {),(}D_{2} {,}\beta_{2,k} {),}...{,(}D_{N} {,}\beta_{N,k} )\} ,(\sum\limits_{n = 1}^{N} {\beta_{n,k} \le 1} {),} \hfill \\ {\text{with rule weight }}\theta_{k} ,k \in \{ 1,2,...,L\} . \hfill \\ and{\text{ attribute weight }}\delta_{1} ,\delta_{2} ,...,\delta_{i} ,i \in \{ 1,2,...,T_{i} \} \hfill \\ in{\text{ C}}_{1} ,{\text{C}}_{2} ,...,{\text{C}}_{n} \hfill \\ \end{gathered}$$

where $x_{1} ,x_{2} ,...,x_{{T_{k} }}$ is the antecedent attribute of the PV power prediction system.$A_{1}^{k} ,A_{2}^{k} ,...,A_{{T_{k} }}^{k}$ is a series of reference values for the antecedent attribute $x_{1} ,x_{2} ,...,x_{{T_{k} }}$.$T_{k}$ is the number of attributes in the kth rule.$D_{1} ,D_{2} ,...,D_{N}$ are the consequences, and $\beta_{1,k} ,\beta_{2,k} ,...,\beta_{N,k}$ are their corresponding belief degrees.$\theta_{k}$ is the weight of the kth belief rule.$\delta_{1} ,\delta_{2} ,...,\delta_{i}$ is the weight of the ith attribute.$L$ is the number of rules.${\text{C}}_{1} ,{\text{C}}_{2} ,...,{\text{C}}_{n}$ is the interpretability constraint of the model. The DBRB-I model is composed of multiple Sub-BRBs in a deep structure. The modelling process of the DBRB-I model based on the PV power generation system is shown in Fig. 1. First, the attributes of PV power generation systems are trended analysed and sorted by correlation to changes in results. Second, the rules of the initial BRB constructed from expert knowledge are simplified through sensitivity analysis. Finally, each Sub-BRBn of DBRB-I is optimized by an optimization algorithm with interpretability.

## The DBRB-I model interpretability

**Figure 2:**
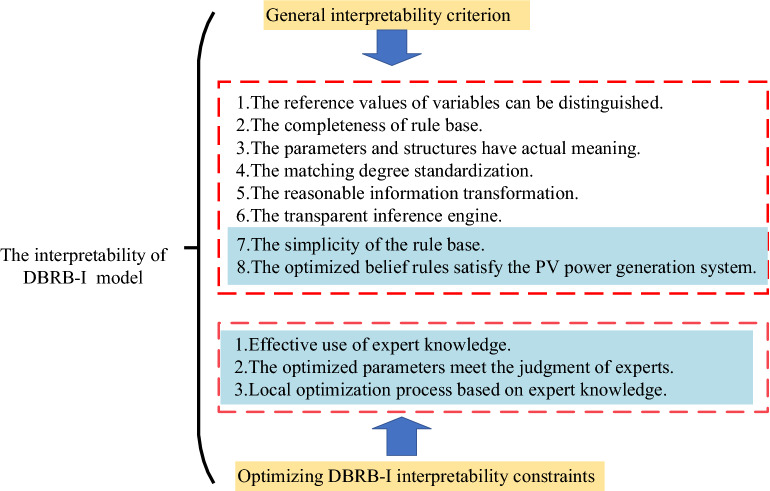
The interpretability of the DBRB-I model.

To maintain the interpretability of the DBRB-I model, it is crucial to construct interpretability constraints for PV power generation systems. Cao et al. constructed a general BRB interpretability criterion^23^. Thus, the DBRB-I model should conform to the general interpretability BRB criterion. Moreover, to make the DBRB-I model more interpretable, this paper focuses on the interpretability criteria7, 8. The interpretability of the DBRB-I model is shown in Fig. 2.

Criterion 7: The simplicity of the rule base.

The modelling method based on IF–THEN rules can make the model structure clear and easy to understand. However, the number of rules grows exponentially in complex systems of PV power generation, which leads to the explosion of rules in the BRB system. Too many rules can reduce the readability of the model and reduce model interpretability.

Thus, to improve the interpretability and readability of the DBRB-I model, the rules in the system should be reasonably reduced. A reasonable number of rules can improve the accuracy of the BRB model^24^. The initial BRB constructed by expert knowledge can be effectively analysed to determine which rules are redundant and which are efficient rules in systems. Therefore, an effective way to reduce rules is to perform sensitivity analysis on the initial BRB model.

Criterion 8: The optimized belief rule satisfies the PV power generation system.

**Figure 3:**
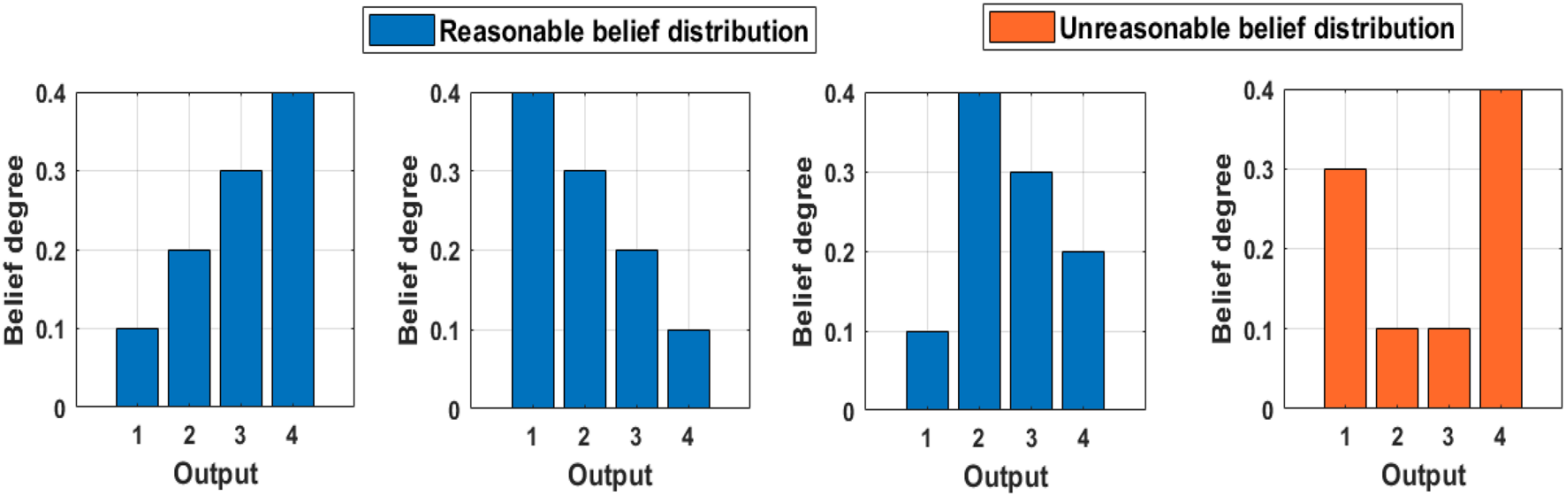
Reasonable and unreasonable belief distributions.

Belief rules can provide a clear semantic description between the input and output of the PV power generation system, which is the main manifestation of the interpretability of the DBRB-I model^25^. Expert knowledge can be introduced into the model as a parameter by belief rules. Thus, the prediction results of the model will be convincing by the expert. However, to obtain better accuracy, the model is optimized in a large search domain. Inevitably, many incorrect rules that are contrary to the actual system will be generated. For example, in the PV power generation predicting system, the belief distribution of the output results are {(Excellent, 0.4353), (Good, 0,0944), (Middle, 0.1098), (Low, 0.3605)}, which means the “Excellent” support of the system's power generation is 0.4353 and the “Low” support is 0.3605. This belief distribution is unrealistic, and a reasonable belief distribution should not give high confidence to conflicting results, as shown in Fig. 3.

Thus, in a PV power generation system, to ensure the reasonableness of the prediction results, the interpretability constraints are as follows:

6 $$\begin{gathered} \beta_{k} \sim U_{k} { (}k = 1,2,...,L{)} \hfill \\ U_{k} \in \{ \{ \beta_{1} \le \beta_{2} \le \cdots \le \beta_{n} \} \hfill \\ \quad or\{ \beta_{1} \ge \beta_{2} \ge \cdots \ge \beta_{n} \} \hfill \\ \quad or\{ \beta_{1} \le \cdots \le max(\beta_{1} ,\beta_{2} ,...\beta_{n} ) \ge \cdots \ge \beta_{n} \} \} \hfill \\ \end{gathered}$$

where $U_{k}$ is the interpretability constraint in the kth rule. For different systems, the interpretability constraints will be different, but it should be noted that the operating mechanism and common sense of the actual system need to be satisfied^25^. Moreover, a reasonable belief distribution shape should be monotonic or convex.

**Figure 4:**
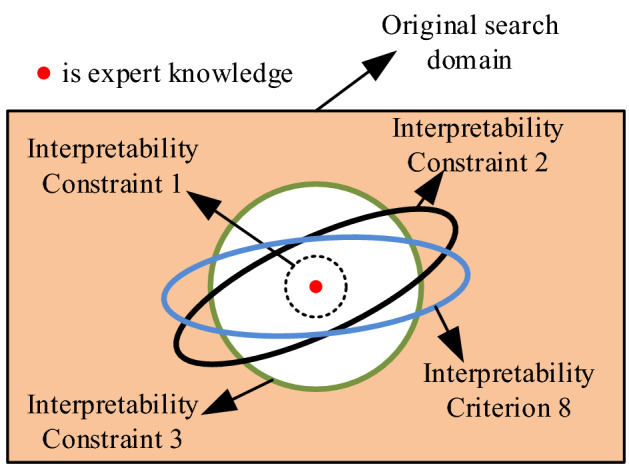
The feasible region of DBRB-I model optimization.

BRB is a rule-based modelling method, and the relationship between the input and output of the model is traceable. Thus, the interpretability of the model structure is the intrinsic feature of BRB. Due to limited expert knowledge, the initial BRB model built by experts does not meet the needs of the actual system, and the model needs to be optimized by observation data. However, optimization algorithms have randomness, which destroys the interpretability of BRB models. Therefore, the following constraints are designed to preserve the interpretability of the BRB model, and the feasible region for DBRB-I model optimization is shown in Fig. 4.

Constraint 1: Effective use of expert knowledge.

Expert knowledge is obtained through the analysis of the actual system and the experience of long-term accumulation. It is one of the important sources of model interpretability^26^. The optimization process of the interpretable BRB model is a local search process based on the initial judgement of experts^22^. Thus, expert knowledge is converted into parameters and brought into the initial population of the optimization algorithm, which can provide guidance for the optimization process and effectively extract useful information from the search space^27^.

7 $$mean^{(g)} = \left\{ {\begin{array}{*{20}c} {Ek,\,\,\,\,\,\,\,\,\,if\,\,\,\,\,\,{\text{ g = 1}}} \\ {mean^{(g)} ,\,\,if\,\,\,\,\,{\text{g}} \ne {1}} \\ \end{array} } \right.$$

Constraint 2: The optimized parameters meet the judgement of experts.

Compared with the black box model, such as the backpropagation neural network (BPNN) and support vector machine (SVM), the parameters of the BRB model are of practical significance, which makes users more trust this model. However, the physical significance of the optimized BRB model parameters may be lost. For example, the initial rule weight is given 0.9 by experts, but the optimized rule weight is 0.005. Experts believe that this rule is crucial, but the results of the model after optimization are inconsistent with the judgment of the expert. This will lead to the reduction of experts' trust in the model. Thus, the key parameters of the BRB model need to be guaranteed to be used effectively. To solve this problem, the parameters of the BRB model are constrained as follows:

8 $$\begin{gathered} P_{lp} \le P \le P_{up} : \{ \theta_{{lp_{k} }} \le \theta_{k} \le \theta_{{up_{k} }} \quad k \in \{ 1,2,...,L\} . \hfill \\ \, \delta_{{lp_{i} }} \le \delta_{i} \le \delta_{{up_{i} }} \quad n \in \{ 1,...,N\} . \hfill \\ \, \beta_{{lp_{{_{k,n} }} }} \le \beta_{{_{{_{k,n} }} }} \le \beta_{{up_{{_{k,n} }} }} \quad i,m \in \{ 1,2,...,T_{k} \} .\} \hfill \\ \end{gathered}$$

where $P_{lp}$ is the minimum value of the parameter and $P_{up}$ is the maximum value of the parameter. Effective constraints for the parameters of the BRB model can avoid modelling accuracy reduction due to overoptimization.

Constraint 3: Local optimization process based on expert knowledge.

The interpretability of the optimization process reflects the optimization in a local search domain judged by experts for interpretability BRBs^22^. Thus, to enhance the interpretability of the model, an interpretability constraint is designed.

Interpretability constraints on individuals of the initial population by introducing Euclidean distance. The Euclidean distance reflects the straight-line distance between two points in space. Constrain the distance between the individual and the expert knowledge of the algorithm, which further realizes the optimization of the local search domain based on the initial judgment of the expert and enhances the interpretability of the optimization.

9 $$\begin{aligned} \rho (x_{n} ,x_{n}^{^{\prime}} ) & = \sqrt {(x_{1} - x_{1}^{^{\prime}} )^{2} + (x_{2} - x_{2}^{^{\prime}} )^{2} + \cdots + (x_{n} - x_{n}^{^{\prime}} )^{2} } \hfill \\ & \quad \rho (x_{n} ,x_{n}^{^{\prime}} ) \le d \hfill \\ \end{aligned}$$

$\rho (x_{n} ,x_{n}^{^{\prime}} )$ is the Euclidean distance between the individuals of the initial population and expert knowledge. $d$ is the parameter of the distance, and the value is determined by the expert.

BRB is a rule-based modeling method that conforms to the method of human knowledge expression. Through expert knowledge and observational data, the reasoning process and modelling process of the BRB model can be easily understood. Moreover, BRB has a transparent reasoning process based on the ER algorithm, and decision makers and users can directly touch and access the model. Thus, interpretability is an intrinsic feature of BRB models. However, any algorithm has randomness, and this randomness destroys the interpretability of the BRB model. Therefore, it is necessary to design the above interpretability constraints to protect the interpretability of the BRB model^23^.

## Sensitivity analysis

An appropriate BRB model structure and parameters are crucial to improve the accuracy and interpretability of the predicted PV output power. In the literature^24^, Zhang et al. demonstrated that a suitable number of rules can improve the accuracy of the BRB model. Moreover, the simplicity of the BRB rule base can improve the readability of the model^23^. Therefore, there are many methods of rule reduction^28,29^. However, reasonable and transparent reduction rules are important for interpretable BRB models. Sensitivity analysis is a great way to simplify the rule base in an interpretable way.

Sensitivity analysis (SA) refers to analysing the direct influence of model input parameters and model results^30^. SA can identify key parameters of the model, which can help users improve the prediction accuracy of the model. There are two main methods of sensitivity analysis: global sensitivity analysis (GSA) and local sensitivity analysis (LSA). GSA refers to the study of the effect of two or more parameters changing together on the model parameters. However, GAS is computationally expensive^30^. LSA refers to exploring changes in the response of a model by changing one parameter of the model while keeping the other parameters constant. The advantages of local sensitivity analysis are simplicity and ease of understanding.

Thus, this paper uses LSA to simplify the structure of the BRB model. The input parameter of the LSA is the rule weight, and the sensitivity of the rule is reflected by the fluctuation of the model error g; that is, the greater the mean square error (MSE), the greater the sensitivity of the rule. Through LSA, users can learn which rules are important and which rules are inefficient. Finally, the inefficient rules are simplified, which simplifies the BRB model and improves the model readability.

10 $$g = \left| {MSE_{SA} - MSE_{{({\text{initial}})}} } \right|.$$

## Reasoning process and optimization process of the DBRB-I model

In “The reasoning process of the DBRB-I model” section, the reasoning process of the DBRB-I model is introduced. Then, in “Optimization process of the DBRB-I model” section, the optimization process of the DBRB-I model is introduced.

### The reasoning process of the DBRB-I model

The reasoning process of BRB is based on the evidential reasoning (ER) algorithm. The ER algorithm with transparency and reliability is described as follows:

- *Step 1* Different forms of input information are transformed into belief distributions.
  11 $$S(x_{i} ) = \{ (A_{i,j} ,\alpha_{i,j} ),i = 1,...,M;j = 1,...,J_{i} \}$$
  12 $$a_{i,j} = \left\{ \begin{gathered} \frac{{A_{i,j + 1} - x_{i} }}{{A_{i,j + 1} - A_{i,j} }}, \, j = k,{\text{ if }}A_{i,j} \le x_{i} \le A_{i,j + 1} \hfill \\ \begin{array}{*{20}l} {\frac{{x_{i} - A_{i,j} }}{{A_{i,j + 1} - A_{i,j} }}, \, j = k + 1 \, } \\ {0, \, j = 1,...,J_{i} ,j \ne k,k + 1} \\ \end{array} \hfill \\ \end{gathered} \right.$$
  where $a_{i,j}$ is the matching degree between the input information and the reference value $A_{i,j}$.
- *Step 2* The activation weight of the kth belief rule is calculated.
  13 $$w_{k} = \frac{{\theta_{k} \prod\limits_{i = 1}^{{M_{k} }} {\left( {a_{i,j}^{k} } \right)} \overline{{^{{\delta_{i} }} }} }}{{\left( {\sum\limits_{l = 1}^{K} {\theta_{l} } \prod\limits_{i = 1}^{{M_{l} }} {(a_{i,j}^{l} )} \overline{{^{{\delta_{i} }} }} } \right)}},\,\,\,\overline{{\delta_{i} }} = \frac{{\delta_{i} }}{{\mathop {max}\limits_{{i = 1,...,M_{k} }} \{ \delta_{i} \} }}$$
- *Step 3* The belief degree of the inference output is generated by the analytical ER algorithm.
  14 $$\beta_{n} = \frac{{\mu \times \left[ {\prod\limits_{i = 1}^{L} {\left( {\omega_{l} \beta_{n,l} + 1 - \omega_{l} \sum\limits_{i = 1}^{N} {\beta_{i,l} } } \right)} - \prod\limits_{l = 1}^{L} {\left( {1 - \omega_{l} \sum\limits_{i = 1}^{N} {\beta_{i,l} } } \right)} } \right]}}{{1 - \mu \times \left[ {\prod\limits_{l = 1}^{L} {\left( {1 - \omega_{l} } \right)} } \right]}}$$
  15 $$\mu = \frac{1}{{\sum\limits_{n = 1}^{N} {\prod\limits_{l = 1}^{L} {\left( {\omega_{l} \beta_{n,l} + 1 - \omega_{l} \sum\limits_{i = 1}^{N} {\beta_{i,l} } } \right) - (N - 1)\prod\limits_{l = 1}^{L} {\left( {1 - \omega_{l} \sum\limits_{i = 1}^{N} {\beta_{i,l} } } \right)} } } }}$$
- *Step 4* The final belief distribution of the inference output is expressed as follows:
  16 $$S(A^{^{\prime}} ) = \{ (D_{n} ,\beta_{n} );n = 1,...,N\}$$
  17 $$u(S(A^{^{\prime}} )) = \sum\limits_{j = 1}^{N} {u(D_{j} )\beta_{j} }$$
  where $A^{^{\prime}}$ is the input vector of the actual system,$u(D_{j} )$ is the utility of the $D_{j}$.$u({\text{S}}(A^{\prime}))$ is the final expected utility.

### Optimization process of the DBRB-I model

In current research, the projection covariance matrix adaptation evolution strategy (P-CMA-ES) optimization algorithm is one of the effective algorithms and has also been applied to the research of different BRBs^23^. The P-CMA-ES optimization algorithm has the following advantages: (1) It has good optimization performance. (2) The algorithm has a fast convergence speed and strong robustness. (3) It has the advantages of rotation invariance and spread rotation invariance. Thus, the P-CMA-ES algorithm is used to optimize the DBRB-I model in this paper.

**Figure 5:**
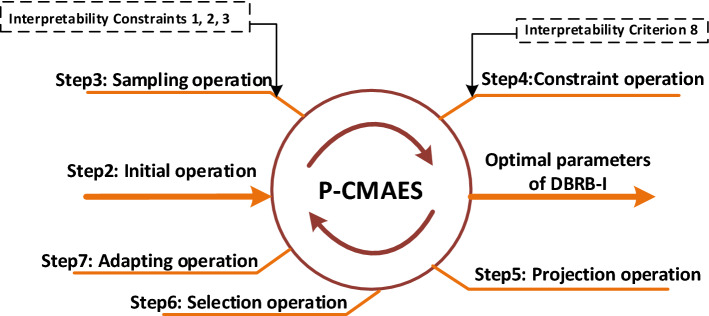
The optimization process of the modified P-CMA-ES.

However, the P-CMA-ES algorithm generates new solutions that will destroy DBRB-I model interpretability. Therefore, to maintain the interpretability of the DBRB-I model, interpretability constraints are added to the original P-CMA-ES algorithm. The newly modified P-CMA-ES optimization algorithm is shown in Fig. 5. The pseudocode of the optimization method is given in Algorithm 1, and the specific process is as follows:

- *Step 1 (Construct the objective function)* To improve the prediction accuracy of the DBRB-I model, the parameters of the model are optimized through the training data. Therefore, the objective optimization function of the DBRB-I model is described as follows:
  18 $$\begin{gathered} \min MSE(\Omega ) \hfill \\ s.t. \, \sum\limits_{n = 1}^{N} {\beta_{{_{{_{k,n} }} }} } = 1 \hfill \\ \, \theta_{{lp_{k} }} \le \theta_{k} \le \theta_{{up_{k} }} \quad k \in \{ 1,2,...,L\} . \hfill \\ \, \delta_{{lp_{i} }} \le \delta_{i} \le \delta_{{up_{i} }} \quad {\text{ n}} \in \{ 1,...,N\} . \hfill \\ \, \beta_{{lp_{{_{k,n} }} }} \le \beta_{{_{{_{k,n} }} }} \le \beta_{{up_{{_{k,n} }} }} \quad i,m \in \{ 1,2,...,T_{k} \} . \hfill \\ \end{gathered}$$
  where $MSE( \cdot )$ is the degree of difference between the predicted value of the PV system and the true value, which can be described as follows:
  19 $$MSE = \frac{1}{n}\sum\limits_{i = 1}^{n} {\left( {y_{i} - \hat{y}_{i} } \right)^{2} }$$
  where $n$ is the number of training data.$y_{i}$ is the true value of the PV power generation system.

$\hat{y}_{i}$ is the predicted value of the DBRB-I model.

- *Step 2 (Initial operation)* Initial population size $\lambda$, the initial mean value $mean^{0} = \Omega^{0} (\theta,\delta ,\beta )$, the offspring population size $\tau$, the initial step size $\varepsilon^{0}$ and the initial covariance matrix $C^{0}$.
- *Step 3 (Sampling operation)* Through interpretability constraints 1, 2, and 3, generate the initial population by
  20 $${\text{Constraints1}}:\,\,\Omega_{k}^{g + 1} = mean^{(g)} + \varepsilon^{(g)} N(0,C^{(g)} ),k = 1,2,...,\lambda$$
  21 $$mean^{(g)} = \left\{ {\begin{array}{*{20}l} {EK, \, if{\text{ g = 1}}} \\ {mean^{(g)},if{\text{ g}} \ne {1}} \\ \end{array} } \right.$$

Interpretability constraint 1 incorporates expert knowledge into the initial population of the model, and expert knowledge can play a guiding role in the model optimization process, which improves the model optimization process. Moreover, interpretability constraint 1 enables the starting point of the optimization to be close to the optimal solution of the model.

22 $${\text{Constraints2}}:\begin{array}{*{20}c} {\Omega_{k}^{(g + 1)} \Leftarrow P = mean^{(g)} + \varepsilon^{(g)} N\left( {0,C^{(g)} } \right)} & {} \\ \begin{gathered} P_{lp} \le P \le P_{up} : \{ \theta_{{lp_{k} }} \le \theta_{k} \le \theta_{{up_{k} }} \hfill \\ \delta_{{lp_{i} }} \le \delta_{i} \le \delta_{{up_{i} }} \hfill \\ \beta_{{lp_{{_{k,n} }} }} \le \beta_{{_{{_{k,n} }} }} \le \beta_{{up_{{_{k,n} }} }} \hfill \\ \end{gathered} & \begin{gathered} \, k \in \{ 1,2,...,L\} . \hfill \\ n \in \{ 1,...,N\} . \hfill \\ i,m \in \{ 1,2,...,T_{k} \} .\} \hfill \\ \end{gathered} \\ \end{array}$$

Interpretability constraint 2 guarantees that the parameters do not lose their physical meaning during optimization, which maintains the interpretability of the model.

23 $${\text{Constraints}}\,{3}:\,\,\begin{array}{*{20}c} {\rho (\Omega,EK) = \sqrt {(x_{1} - x_{1}^{^{\prime}} )^{2} + (x_{2} - x_{2}^{^{\prime}} )^{2} + \cdots + (x_{n} - x_{n}^{^{\prime}} )^{2} } } \\ {\rho (x_{n},x_{n}^{^{\prime}} ) \le d} \\ \end{array}$$

Interpretability constraint 3 guarantees that the optimization process is a local optimization based on expert knowledge for the interpretability model, which further enables the optimized parameters to have good similarity to the expert knowledge.

- *Step 4 (Constraint operation)* Through interpretability criterion 8, the rules that do not meet the actual system are adjusted.
  24 $$\begin{gathered} \Omega_{k}^{(g + 1)} \Leftarrow \beta_{k}^{(g + 1)} = mean^{(g)} + \varepsilon^{(g)} N(0,C^{(g)} ) \hfill \\ \beta_{k}^{(g + 1)} \sim C_{8},k = 1,2,...,\lambda \hfill \\ \end{gathered}$$
  where $\Omega_{k}^{(g + 1)}$ is the kth solution in the (g + 1)th generation, which may not satisfy the belief distribution of the actual system.$\beta_{k}^{(g + 1)}$ is the newly generated belief distribution satisfying the interpretability criterion 8.$\Leftarrow$ is the replacement operation.
- *Step 5 (Projection operation)* To satisfy the equality constraint, the projection operation maps candidates back into the feasible region hyperplane.
  25 $$A_{e} \Omega_{k}^{(g + 1)} (1 + n_{e} \times (j - 1):n_{e} \times j) = 1,j = 1,2,...,N + 1$$

The projection operation is implemented as follows:

26 $$\begin{gathered} \Omega_{k}^{(g + 1)} (1 + n_{e} \times (j - 1):n_{e} \times j) = \Omega_{k}^{(g + 1)} (1 + n_{e} \times (j - 1):n_{e} \times j) \hfill \\ - A_{e}^{T} \times (A_{e} \times A_{e}^{T} )^{ - 1} \times \Omega_{k}^{(g + 1)} (1 + n_{e} \times (j - 1):n_{e} \times j) \times A_{e} \hfill \\ \end{gathered}$$

- *Step 6 (Selection operation)* Calculate the MSE value of population individuals and sort them. The process is described as follows:
  27 $$MSE(\Omega_{ \, 1:\lambda }^{(g + 1)} ) \le MSE(\Omega_{ \, 2:\lambda }^{(g + 1)} ) \le \cdots \le MSE(\Omega_{ \, i:\lambda }^{(g + 1)} ) \le \cdots \le MSE(\Omega_{ \, \lambda :\lambda }^{(g + 1)} )$$

The optimal subgroup is updated as follows:

28 $$mean^{(g + 1)} = \sum\nolimits_{i = 1}^{\mu } {\omega_{i} \Omega_{ \, i:\lambda }^{(g + 1)} }$$

- *Step 7 (Adapting operation)* Update the search covariance matrix, the evolution path of the covariance matrix, the search step size and the evolution path through the most subgroup strategy.
  29 $$\begin{aligned} C^{(g + 1)} & = (1 - c_{1} - c_{2} ) \cdot C^{(g)} + c_{1} p_{c}^{(g + 1)} \left( {p_{c}^{(g + 1)} } \right)^{T} \\ & \,\,\,\, + c_{2} \sum\limits_{i = 1}^{\tau } {\omega_{i} } \left( {\frac{{\Omega_{ \, i:\lambda }^{(g + 1)} - mean^{(g)} }}{{\varepsilon^{(g)} }}} \right)\left( {\frac{{\Omega_{ \, i:\lambda }^{(g + 1)} - mean^{(g)} }}{{\varepsilon^{(g)} }}} \right)^{T} \\ \end{aligned}$$
  30 $$\begin{aligned} p_{c}^{(g + 1)} & = (1 - c_{c} ) \cdot p_{c}^{(g)} + \sqrt {c_{c} (2 - c_{c} )} \cdot \left( {\sum\nolimits_{i = 1}^{\tau } {w_{i}^{2} } } \right)^{ - 0.5} \\ \quad\,\,\,\,\, \cdot \left( {{\text{mean}}^{(g + 1)} - {\text{mean}}^{(g)} } \right)/\varepsilon^{(g)} \\ \end{aligned}$$
  31 $$\varepsilon^{g + 1} = \varepsilon^{g} \exp \left( {\frac{{c_{\sigma } }}{{d_{\sigma } }}\left( {\frac{{\left\| {p_{ \, \sigma }^{g + 1} } \right\|}}{{E\left\| {N(0,1)} \right\|}} - 1} \right)} \right)$$
  32 $$\begin{gathered} p_{\sigma }^{(g + 1)} = (1 - c_{c} ) \cdot p_{ \, \sigma }^{(g)} + \sqrt {c_{c} (2 - c_{c} )} \cdot \left( {\sum\nolimits_{i = 1}^{\tau } {w_{i}^{2} } } \right)^{ - 0.5} \hfill \\ \quad\, \cdot \left( {C^{(g)} } \right)^{ - 0.5} \cdot \left( {mean^{(g + 1)} - mean^{(g)} } \right)/\varepsilon^{(g)} \hfill \\ \end{gathered}$$
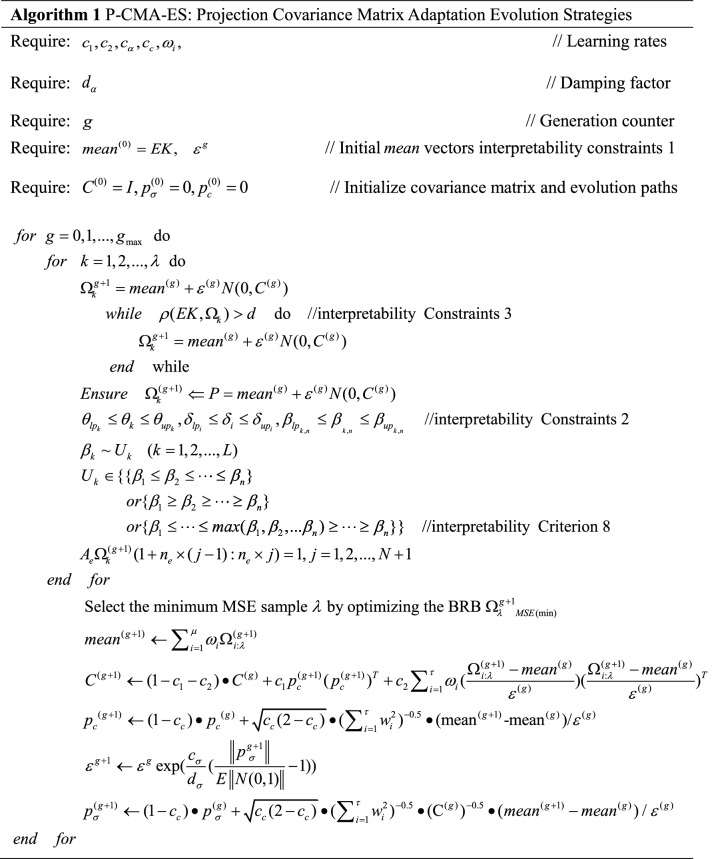

## Case study

In “Description of the dataset” section, the dataset is described. The initial DBRB-I model is constructed in “Construct the initial BRB by expert knowledge” section. Then, in “Sensitivity analysis of the initial DBRB-I model” section, a sensitivity analysis of the rules of the DBRB-I model is presented. The optimized DBRB-I model is described in “The optimized DBRB-I model” section. In “Discussion of the interpretability of the DBRB-I model” section, the interpretability of the DBRB-I model is discussed.

The increase in the proportion of PV power generation will increase the difficulty of power grid scheduling. When the proportion exceeds 15%, it may cause paralysis of the grid system^31^. An interpretable model can provide grid operators with some reference for grid scheduling and management. Thus, it is very important to predict PV power in a reliable, safe and interpretable way^31^.

### Description of the dataset

**Figure 6:**
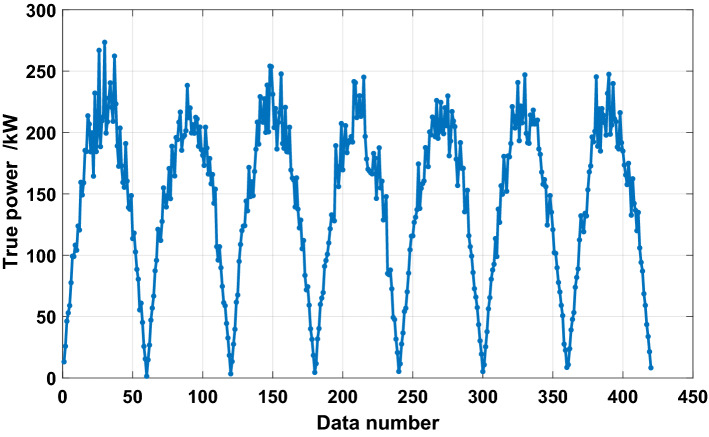
Data observation.

The PV power dataset is obtained from AI Studio. The dataset is the operation data of PV modules in China in 2018. The data collection interval was 10 min, and the data were desensitized. This paper selects a week of data from January 1, 2018, to January 7, 2018, for the experiment, as shown in Fig. 6. A total of 280 data samples are used for training, and 140 data samples are used for testing.

### Construct the initial BRB by expert knowledge

**Figure 7:**
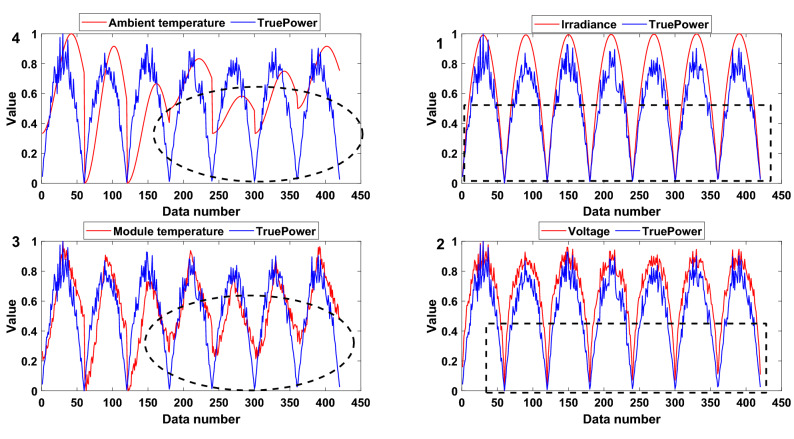
The relationship between each attribute and output power.

**Figure 8:**
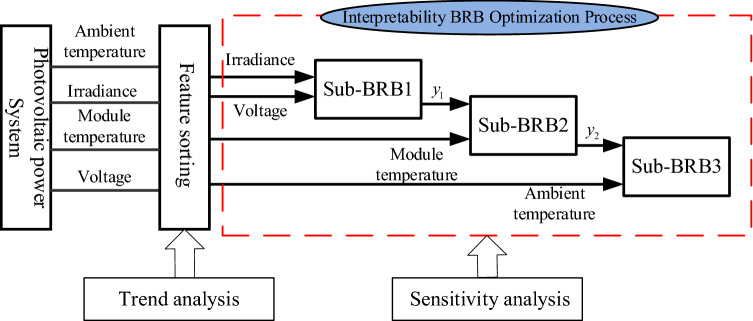
Initial PV output power prediction model based on DBRB-I.

PV power generation systems use solar energy to generate electricity, and their output power is strongly affected by solar irradiance. At the same time, voltage, ambient temperature and module temperature are important factors that affect PV output efficiency^32^. Then, the dataset is normalized, and the relationship between each attribute and output power is trend analysed, as shown in Fig. 7. The irradiance has the largest relationship with the output power, while the ambient temperature has the smallest relationship with the output power, and the most obvious part is marked with a black curve. Thus, the order of importance to the output power attributes of the PV power generation system is as follows: irradiance, voltage, module temperature, and ambient temperature. The initial DBRB-I model is shown in Fig. 8.

In practical engineering, the selection of the reference value requires the judgment of expert knowledge to select the interval range with typical significance and then combine the data statistics to give accurate results^33^. Thus, normalized data usually use four semantic values to describe the attributes and the state of the system, that is, “Excellent”, “Good”, “Middle”, and “Low”. The reference values are given in Tables 1 and 2. Moreover, the beliefs of the initial models Sub-BRB1, Sub-BRB2 and Sub-BRB3 are shown in “Appendix A”.

**Table 1:** The attribute reference value.

Attribute	Attribute weight	Excellent	Good	Middle	Low
Irriadiance	1	1	0.8	0.4	0
Voltage	1	1	0.8	0.4	0
Module temperature	1	1	0.8	0.4	0
Ambient temperature	1	1	0.8	0.4	0

**Table 2:** The output power of the PV power generation system.

Referential points	Excellent	Good	Middle	Low
Referential value	1	0.8	0.4	0

### Sensitivity analysis of the initial DBRB-I model

**Figure 9:**
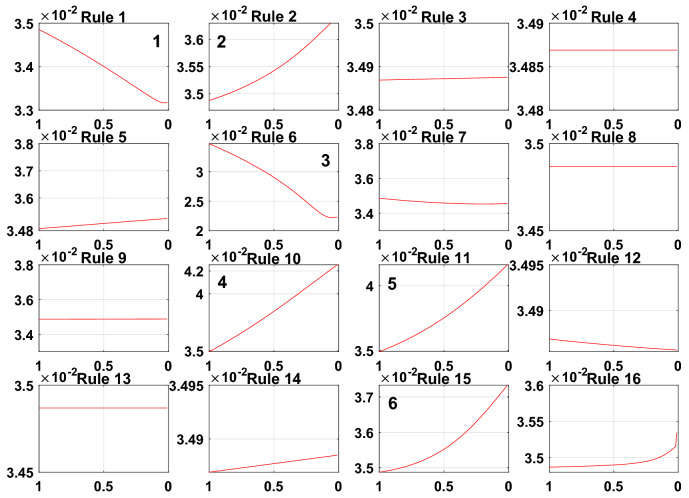
The sensitivity analysis for Sub-BRB1 (x-axis represents rule weights, y-axis represents MSE).

**Figure 10:**
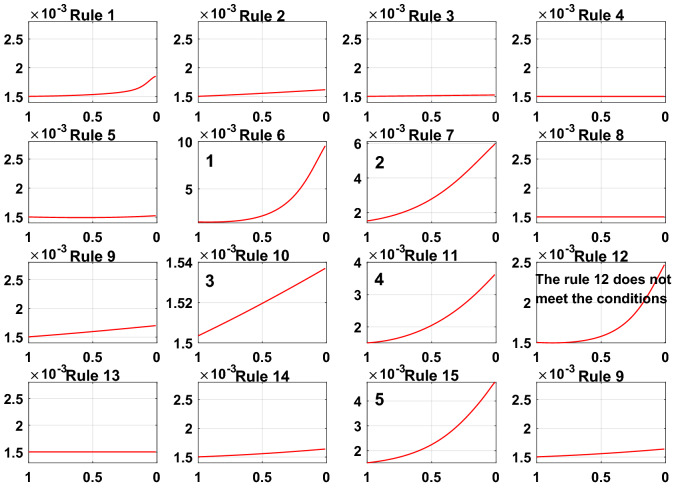
The sensitivity analysis for Sub-BRB2 (x-axis represents rule weights, y-axis represents MSE).

**Figure 11:**
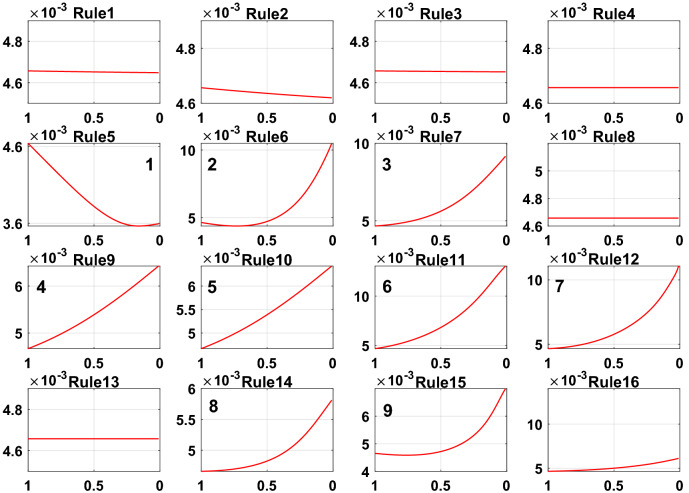
The sensitivity analysis for Sub-BRB3 (x-axis represents rule weights, y-axis represents MSE).

To meet the needs of the PV power generation system, the model error g = 0.001. A sensitivity analysis of the rules for the initially constructed Sub-BRB1, Sub-BRB2 and Sub-BRB3 is shown in Figs. 9, 10, and 11, respectively. Rules 3, 4, 5, 7, 8, 9, 12, 13, 14, and 16 in Sub-BRB1 have little effect on the system. These rules can be considered inefficient rules of the system. However, rules 1, 2, 6, 10, 11, and 15 have a large impact on the system and satisfy the model error g.

The purpose of sensitivity analysis is to remove inefficient rules, which will reduce the complexity of the model and increase the readability of the model by describing the PV system with fewer rules. Moreover, the difficulty of optimization is reduced, and the effect of optimization is improved. Table 3 shows the effective rules of each sub-BRB of DBRB-I.

**Table 3:** The number of DBRB-I model rules.

	Number of initial rules	Number of efficient rules	Efficient rule
Sub-BRB1	16	6	Rule: 1,2,6,10,11,15
Sub-BRB2	16	5	Rule: 6,7,10,11,15
Sub-BRB3	16	9	Rule: 5,6,7,9,10,11,12,14,15

**Figure 12:**
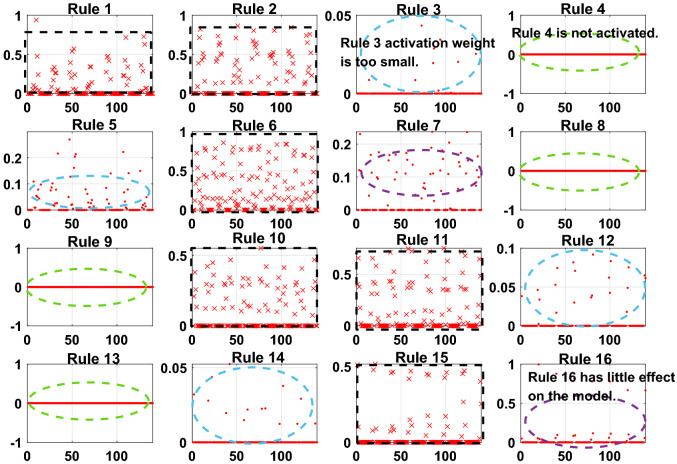
Analysis of Sub-BRB1 rule activation weights (x-axis represents test data, y-axis represents activation weights).

**Figure 13:**
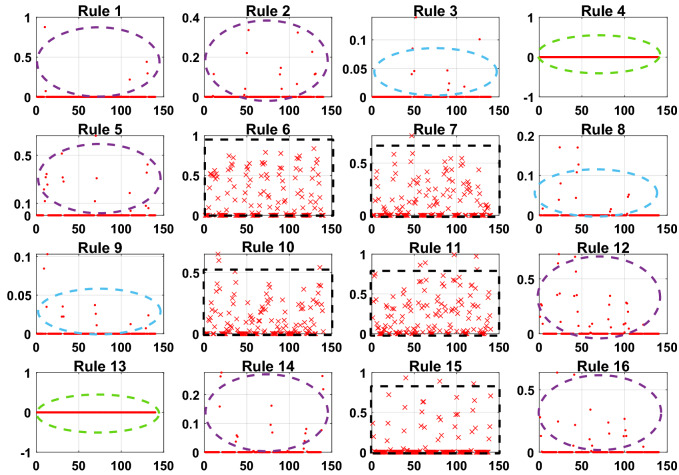
Analysis of Sub-BRB2 rule activation weights (x-axis represents test data, y-axis represents activation weights).

**Figure 14:**
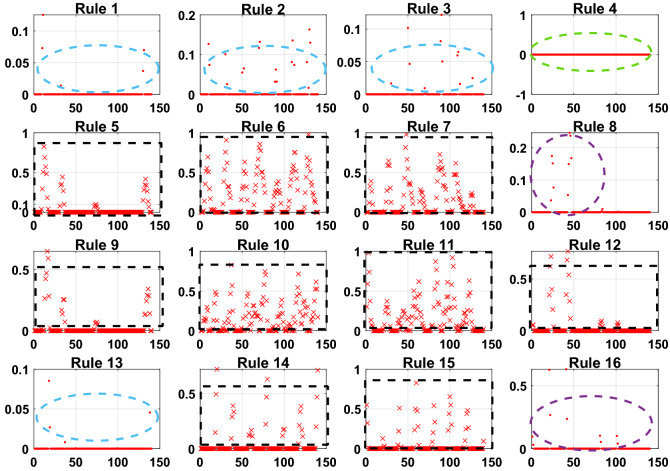
Analysis of Sub-BRB3 rule activation weights (x-axis represents test data, y-axis represents activation weights).

To further prove the redundancy of inefficient rules, the rule activation weights of DBRB-I are analyzed. Figures 12, 13, and 14 show the activation weights of each rule for Sub-BRB1, Sub-BRB2, and Sub-BRB3, respectively. As seen from Fig. 11 (the blue curve represents that the activation weight of the rule is too small, the green curve represents that the rule is not activated, the black curve represents that the rule is very important, and the purple curve represents that the rule has little effect on the model), the activation weight of rules 3, 12, and 14 is too small, it is difficult for experts to judge whether rule 3 has an effect on the system, which reduces the user's understanding of the model, and rule 3 will reduce the interpretability of the model. Thus, rule 3 is classified as an inefficient rule. Moreover, rules 4, 8, 9, and 13 have no effect on the model, which shows that these rules are redundant. Reducing these rules does not affect the accuracy of the model but improves the interpretability of the model. The number of times rule 6 is activated in the system shows that rule 6 is very important to the system. Rule 6 is activated many times in the system, which shows that rule 6 has a great influence on the system. Finally, although the activation weight of rule 16 is relatively large, the number of activations is too small, and it has little effect on the model, so rule 16 is also classified as an inefficient rule.

**Figure 15:**
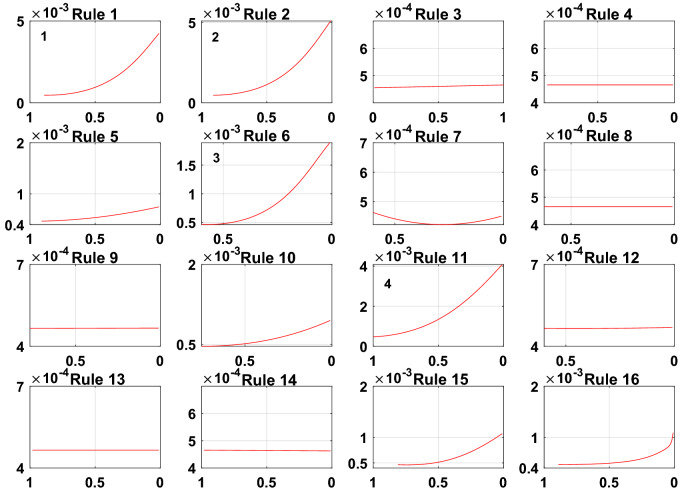
The sensitivity analysis of trained Sub-BRB1 (x-axis represents rule weights, y-axis represents MSE).

**Figure 16:**
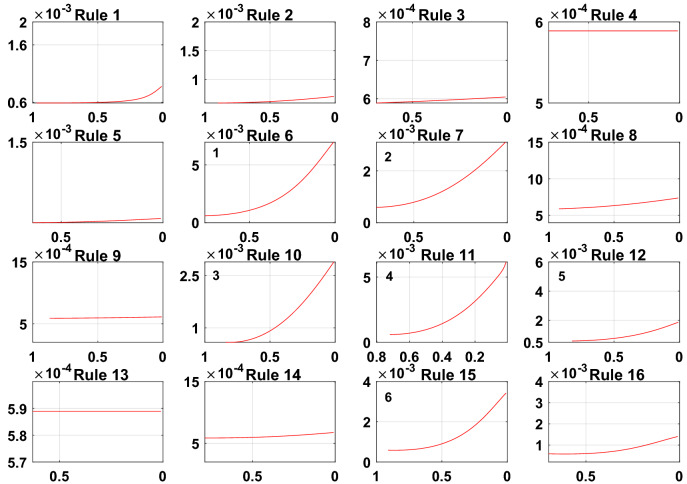
The sensitivity analysis of trained Sub-BRB2 (x-axis represents rule weights, y-axis represents MSE).

**Figure 17:**
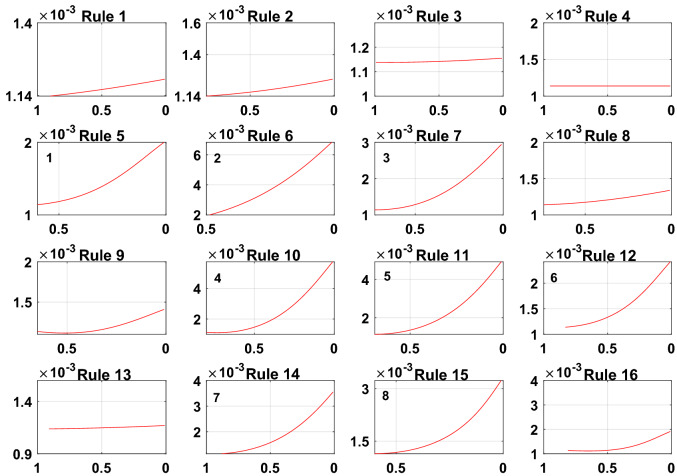
The sensitivity analysis of trained Sub-BRB3 (x-axis represents rule weights, y-axis represents MSE).

To prove the reliability and rationality of expert knowledge and whether the DBRB-I model can correctly reduce inefficient rules, the sensitivity analysis of the Sub-BRB1, Sub-BRB2 and Sub-BRB3 rules after DBRB-I model training is shown in Figs. 15, 16 and 17, respectively. Table 4 gives the effective rules for the trained DBRB-I model to satisfy the threshold g. Through the comparison of Tables 3 and 4, the inefficient rules of the DBRB-I model after training are consistent with the judgment of expert knowledge. Reasonable rule reduction for the initial DBRB-I can reduce the complexity of the model and improve the optimization process of the model. In this paper, the initial DBRB-I is constructed from expert knowledge, and the expert knowledge is reliable. Only rule 12 in Sub-BRB2 is inconsistent with the judgment of the initial DBRB-I model. However, in Fig. 10, rule 12 has a certain effect on the initial model but does not meet the threshold g. Moreover, as shown in Fig. 13, the partial activation weight of rule 12 is too small, and experts cannot judge whether this rule works, which will reduce the interpretability of the model. Therefore, rule 12 is considered an inefficient rule in the initial model.

**Table 4:** The number of trained DBRB-I model rules.

	Number of initial rules	Number of efficient rules	Efficient rule
Sub-BRB1	16	4	Rule: 1,2,6,11
Sub-BRB2	16	6	Rule: 6,7,10,11,12,15
Sub-BRB3	16	8	Rule: 5,6,7,10,11,12,14,15

### The optimized DBRB-I model

In this paper, for interpretability criterion 8 and interpretability constraint 1,2, as shown in “Appendix A”, the d of interpretability constraint 3 is 1.4, 1.4, 1.9, respectively. The DBRB-I model with 20 rules is named DBRB-I(20), the DBRB-I model with 41 rules is named DBRB-I(41), and the DBRB-I model without interpretability constraints is named DBRB. The optimized parameters of the DBRB-I(20) model are shown in “Appendix B”.

In “Transparency and traceability of the DBRB-I model” section, transparency and traceability of the DBRB-I model are demonstrated. The DBRB-I model can meet the judgment of experts in “The DBRB-I model conforms to expert judgment” section. Then, in “Rule reduction under different conditions” section, the rule reductions under different threshold g are given. The interpretability constraints of the optimized DBRB-I model are analyzed in “Analysis of interpretability constraints of the DBRB I model” section. In “Analysis of convergence of the DBRB I model” section, the convergence of the DBRB-I model is analyzed. The accuracy of the DBRB-I model is analyzed in ’Analysis of the accuracy of different DBRB I models“ section. Then, in “Robustness Analysis of the DBRB I model” section, the robustness of the DBRB-I model is analyzed. Skill scores are calculated in “Analysis of DBRB-I model skill scores” section.

#### Transparency and traceability of the DBRB-I model

**Figure 18:**
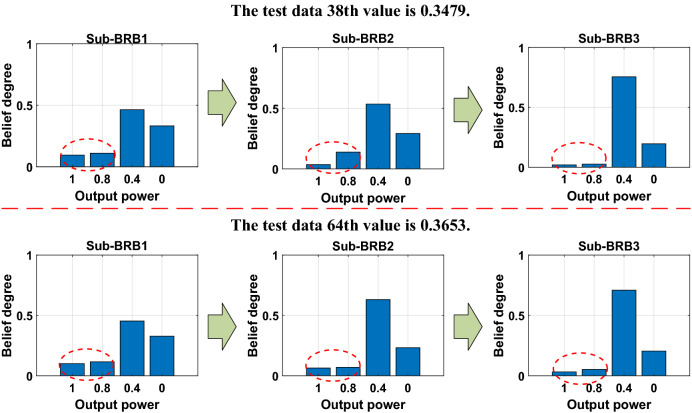
Transparency and validity of the DBRB-I(20) model.

The transparency and traceability of the DBRB-I(20) model are shown in Fig. 18. Taking the 38th and 64th test data as an example, the true values of the 38th and 64th test data are 0.3479 and 0.3653, respectively. In the DBRB-I(20) model, the belief level of 0.4 is continuously improved, while the other belief levels continuously lower, which is practical and understandable by the user. This shows that the DBRB-I(20) model can continuously improve the accuracy of the model. Moreover, every test data point is a clear, transparent and traceable process.

#### The DBRB-I model conforms to expert judgment

**Figure 19:**
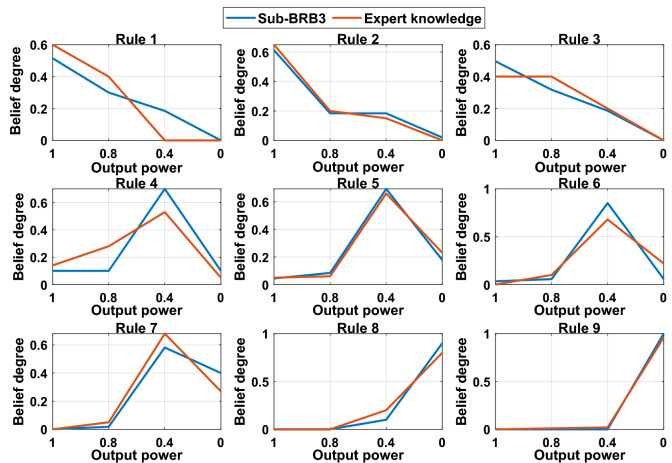
The belief distribution of Sub-BRB3.

The belief distribution of each rule of Sub-BRB3 is shown in Fig. 19. In rules 2, 3, 5, 6, 7, 8, and 9, the optimized belief distribution is close to the expert knowledge, which shows that Sub-BRB3 improves accuracy while maintaining interpretability. In rules 1 and 4, the overall belief distribution of each rule is consistent with the actual system and can be trusted by users. Moreover, it can be seen that the belief distributions in the optimized DBRB-I(20) are close to the initial DBRB-I(20), which indicates that these parameters are locally optimized based on the initial judgement of experts.

#### Rule reduction under different conditions

**Figure 20:**
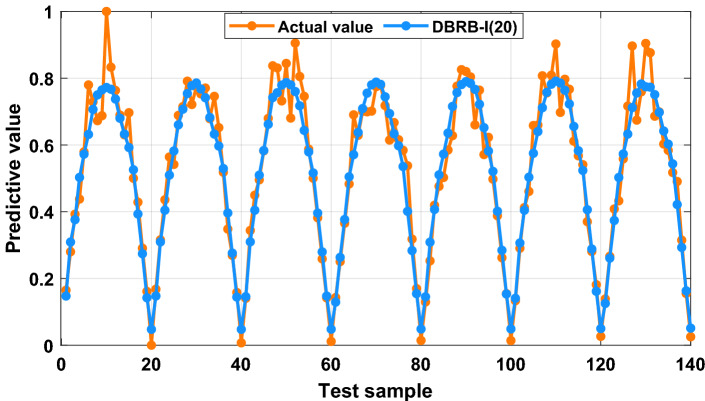
Prediction results of the DBRB-I(20) model.

The prediction results of the DBRB-I(20) model are shown in Fig. 20. The DBRB-I(20) model can predict the entire change trend of the PV power generation system, but it does not have the ability to accurately predict the output result of “excellent”. The reason for the poor prediction effect is that the rules with less influence of the model are simplified. An effective way to improve the accuracy of the DBRB-I(20) model is to lower the threshold g for rule reduction and allow more rules to participate in the model.

**Figure 21:**
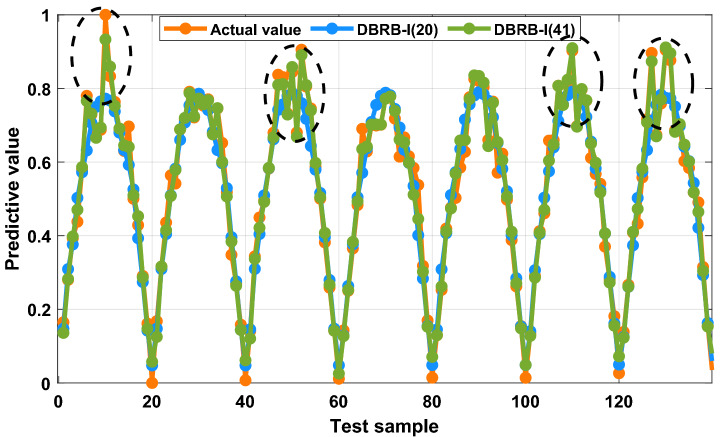
Prediction results of the DBRB-I(41) model.

To prove that reducing the rule reduction threshold g can improve the accuracy of the model, therefore, the threshold g = 0, only the rules that have no effect on the system are reduced for the DBRB-I model. Table 5 gives the number of rules for DBRB-I(41). As shown in Fig. 21, the DBRB-I(41) model can accurately predict the PV power generation system in an interpretable manner. However, DBRB-I(41) has 41 rules, and DBRB-I(20) has 20 rules; that is, the DBRB-I(20) model is more readable than DBRB-I(41).

**Table 5:** The DBRB-I model rules.

Model		Number of rules	Effective rules
DBRB-I(41) And DBRB(41)	Sub-BRB1	12	Rule 1,2,3,5,6,7,10,11,12,14,15,16
	Sub-BRB2	14	Rule:1,2,3,5,6,7,8,9,10,11,12,14,15,16
	Sub-BRB3	15	Rule 1,2,3,5,6,7,8,9,10,11,12,13,14,15,16

**Figure 22:**
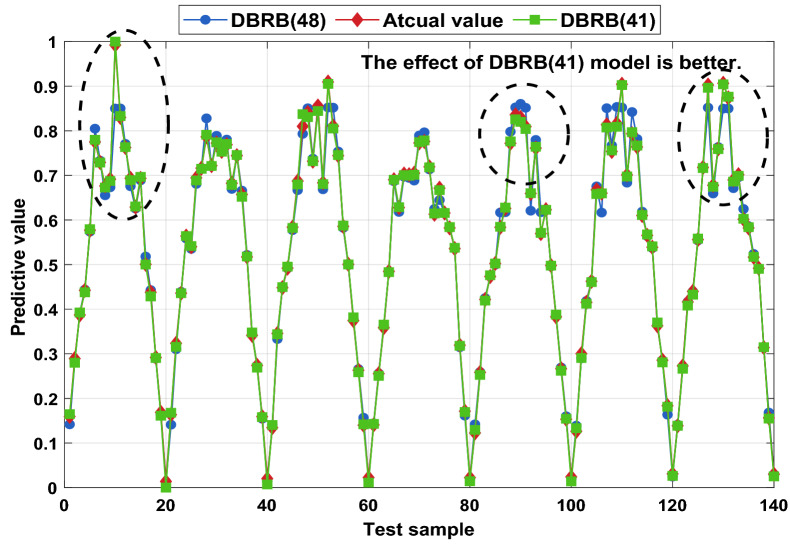
Comparison of DBRB(41) and DBRB(48).

To further prove that removing inefficient rules can improve the accuracy of the model, a comparison between the DBRB(48) model and the DBRB(41) model is shown in Fig. 22. There are 7 fewer rules and 35 fewer optimization parameters in the entire optimization process, which saves more computing resources for DBRB(41) and improves the optimization process of the DBRB(41) model. The MSEs of DBRB(41) and DBRB(48) are 2.81E-5 and 4.83E-4, respectively. Figure 22 shows the comparison between DBRB(41), DBRB(48) and the atcual value. It can be seen that the prediction effect of DBRB(41) is better than that of DBRB(48). This proves that reducing inefficient rules can improve the accuracy of the model. When inefficient rules exist in the system, it will increase the burden on the optimization process of the model and reduce the interpretability of the model. Therefore, choosing appropriate rules is crucial for the model.

#### Analysis of interpretability constraints of the DBRB-I model

**Figure 23:**
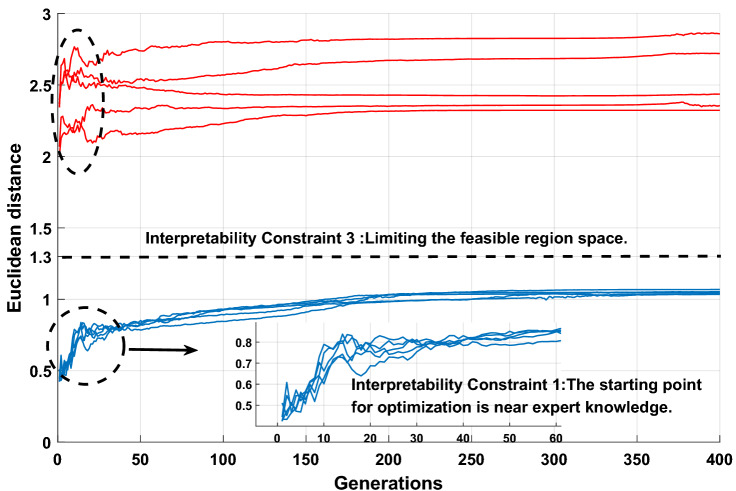
Interpretability analysis of Sub-BRB3.

The effectiveness of interpretability constraints 1 and 3 is demonstrated in Fig. 23. The blue and red curves are the modified P-CMAES algorithm and the original P-CMAES algorithm, respectively. Compared with the original P-CMAES algorithm, the modified P-CMAES algorithm is more suitable for the optimization process of the interpretability model. The starting point of the optimization process of the modified algorithm starts from the vicinity of the expert knowledge so that the initial population of the algorithm will carry some characteristics of the expert knowledge information. Moreover, the optimization process of the DBRB-I model is a local search process based on the initial judgment of expert knowledge. This proves that the optimization process of the modified algorithm can maintain a good balance between model interpretability and modelling accuracy.

For the DBRB-I model, the expert knowledge accumulated from the PV power generation system plays a crucial role in the interpretability of the model. The parameters obtained by expert knowledge are closer to the optimal point in the feasible domain search space; that is, expert knowledge can provide a guiding direction for the optimization process. Therefore, putting expert knowledge into the initial population of the algorithm can improve the optimization process of the model. Furthermore, interpretability constraint 3 further realizes that the optimization process of the DBRB-I model is a local search domain based on expert judgment.

Belief rule base is an intelligent expert system that combines the characteristics of expert system and data-driven model^21^. Experts can build the initial BRB model according to their experience and domain knowledge, which effectively reduces the difficulty of parameter optimization and improves the interpretability of the model^23^. Moreover, the model parameters are further optimized by observation data, which enables the BRB model to achieve good accuracy. Thus, the DBRB-I model is a hybrid model that combines knowledge and data. The initial DBRB-I model is reasonably constructed by expert knowledge, so that the model has good interpretability. Through observational data, an optimization algorithm with interpretability constraints is used for optimization, enabling fine-tuning of parameters based on expert knowledge. Therefore, the DBRB-I model is able to maintain a good balance between interpretability and accuracy.

#### Analysis of convergence of the DBRB-I model

**Figure 24:**
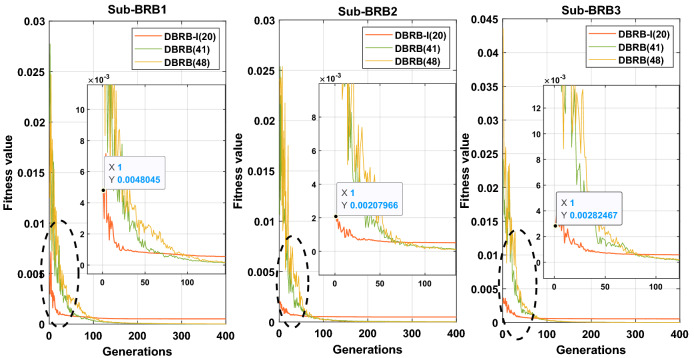
The fitness value of the DBRB model over 400 generations.

The convergence rate of the DBRB-I(20) model is shown in Fig. 24, which shows that DBRB-I (20) has a faster convergence rate and the starting point of the optimization is closer to the optimal solution. The reasons for this phenomenon are as follows: First, DBRB-I(20) reduces the inefficient rules and improves the optimization process of the model. Second, the DBRB-I(20) model has interpretability constraints that limit the solution space. Moreover, DBRB-I(20) can make the starting point of the optimization process close to the optimal solution through interpretability constraint 1. However, the model convergence accuracy is limited due to the reduction of inefficiency rules and the increase of interpretability constraints for the DBRB-I(20) model. Moreover, DBRB(41) reduces redundant rules in the model and reduces the complexity of the model, so DBRB(41) converges faster than DBRB(48). Due to the use of multiple interpretability constraints in the optimization process, the model has good performance in interpretability and accuracy, but the DBRB-I model shows a longer execution time, as shown in Appendix Table B4.

#### Analysis of the accuracy of different DBRB-I models

**Figure 25:**
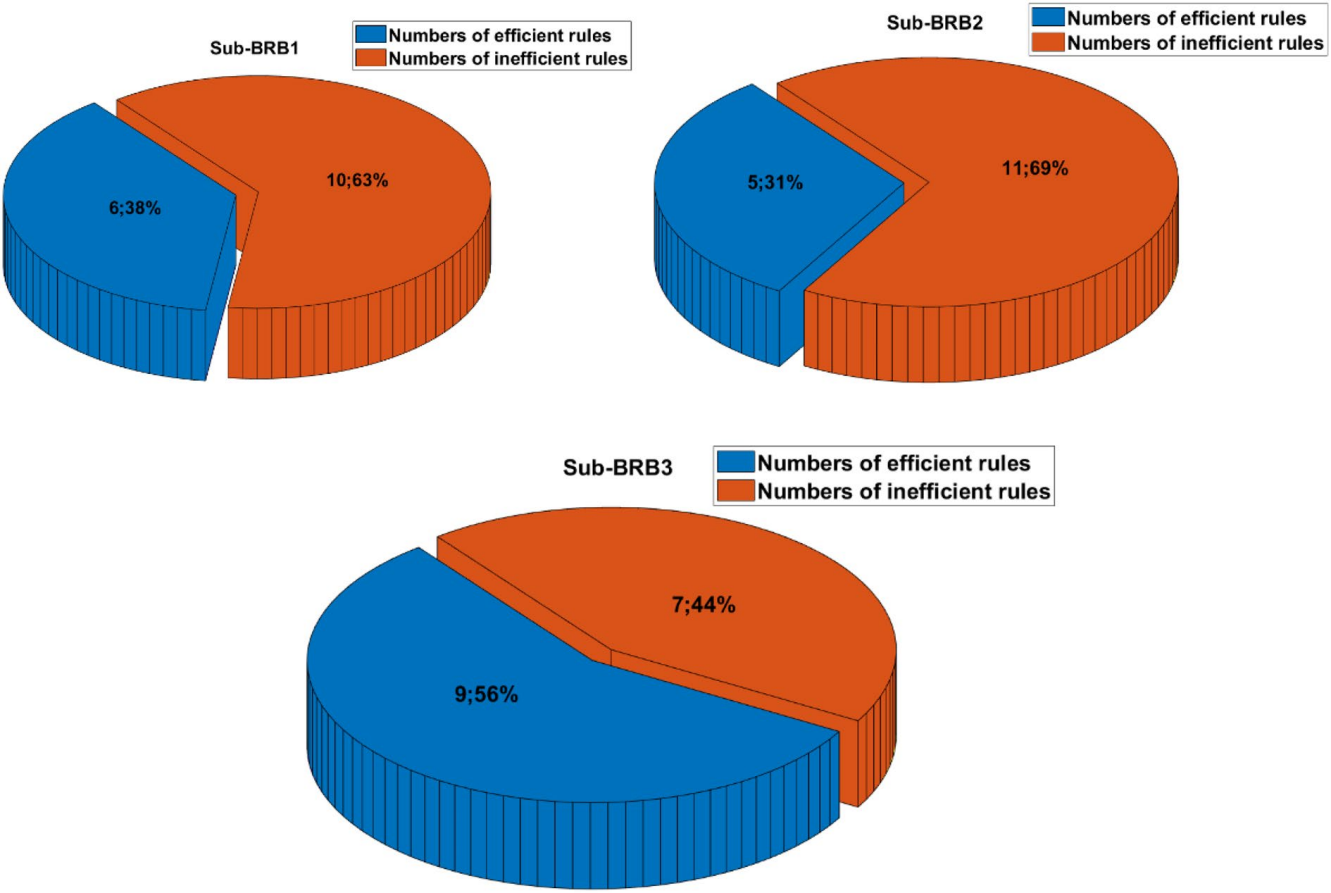
Comparison of the number of effective rules of the DBRB-I(20) model.

Figure 25 shows the comparison between the DBRB-I(20) model and the number of rules per sublayer of DBRB(48). Table 6 shows the comparison of the DBRB-I model with other models. Although the prediction accuracy of the DBRB-I(20) model is not as good as that of DBRB(48), the DBRB-I(20) model is interpretable. The interpretability of DBRB-I(20) is as follows: (1) The belief distribution of the optimized model satisfies the PV power generation system, while DBRB(48) has no such capability. (2) The optimization of the DBRB-I(20) model is carried out in the local search domain judged by experts, which indicates that the optimized parameters of the DBRB-I(20) model are fine-tuned on the basis of expert knowledge; that is, the DBRB-I(20) model can be used in interpretability and precision are well balanced. However, DBRB(48) is optimized in the entire solution space, and the optimized parameters will conflict with expert knowledge. 3) The number of rules for the DBRB-I(20) model is 20, while the number of rules for DBRB(48) is 48. The DBRB-I(20) model reduces the complexity of the system by using fewer rules, the readability of the model is improved, and the interpretability of the model is enhanced.

**Table 6:** Comparison of model accuracy.

Model	Number of rule	MSE
DBRB-I(20)	20	8.17E-4
DBRB-I(41)	41	7.05E-4
DBRB-I(48)	48	7.63E-4
DBRB(41)	41	2.81E-5
DBRB(48)	48	4.83E-4

#### Robustness analysis of the DBRB-I model

**Figure 26:**
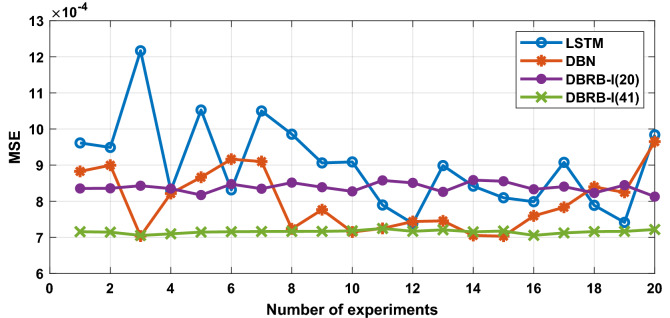
Compare predictions from different models.

A comparison of the prediction results of the DBRB-I model, the popular long short-term memory (LSTM) model and the deep boltzmann machine (DBN) model is shown in Fig. 26^34–36^. To verify the robustness of the proposed DBRB-I model, 20 repeated optimization processes are conducted. Although the accuracy of the DBRB-I model is similar to that of LSTM and DBN, the DBRB-I model is an interpretable model, while LSTM and DBN have no such ability. Moreover, the robustness analysis of the model is shown in Table 7. The MSE standard deviation of DBRB-I is smaller than that of LSTM and DBN, which means the robustness of the DBRB-I model is stronger than LSTM and DBN. In practical engineering, the DBRB-I model is more suitable for reliable and safe systems^23^.

**Table 7:** Robustness analysis of the model.

Model	Max MSE	Min MSE	Average MSE	The standard deviation of MSE
DBRB-I(20)	8.58E−4	8.12E−4	8.38E−4	1.30E−5
DBRB-I(41)	7.25E−4	7.05E−4	7.15E−4	4.67E−6
LSTM	1.21E−3	7.38E−4	8.99E−4	1.20E−4
DBN	9.65E−4	7.03E−4	8.01E−4	8.32E−5

#### Analysis of DBRB-I model skill scores

To further verify the prediction effect of the DBRB-I model, benchmarking is also performed by skill score, which is defined as follows^37^:

33 $$Skill\,\,score = 1 - \frac{{error_{proposed} }}{{error_{reference} }}$$

where $error_{proposed}$ is the error of the proposed model and $error_{reference}$ is the error of the reference model.

Through the above analysis, when the reference model selects the LSTM model, the DBRB-I (41) skill score = 0.20; when the reference model selects the DBN model, the DBRB-I(41) skill score = 0.11. Although the accuracy improvement of the DBRB-I(41) model is not large, the DBRB-I(41) model is an interpretable model. The interpretability of the DBRB-I model compared with LSTM and DBN is as follows: (1) The DBRB-I model is a rule-based modeling method, which can describe the modeling process of the system in a language semantic way. (2) The DBRB-I model has a transparent inference engine, which makes the internal structure clear and transparent and can be directly accessed by users. (3) The DBRB-I model can incorporate expert knowledge and system mechanisms, which can better help people understand and trust the model.

### Discussion of the interpretability of the DBRB-I model

**Figure 27:**
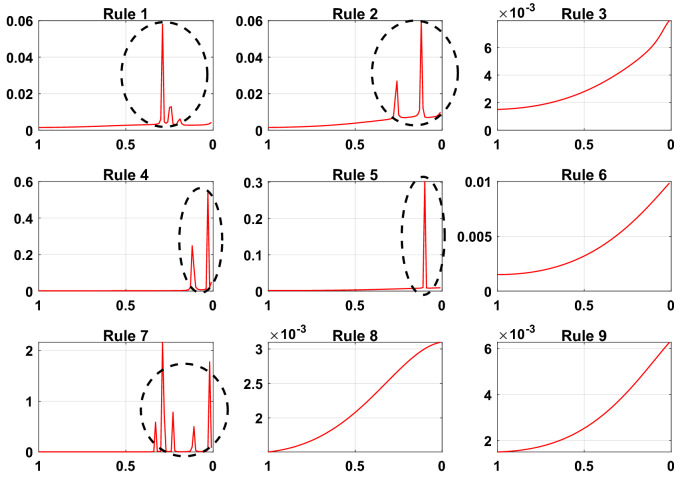
Sensitivity analysis of Sub-BRB3 of the DBRB-I(20) model (x-axis represents rule weights, y-axis represents MSE).

As an interpretable model, DBRB-I has the characteristics of a transparent reasoning process, process interpretability, and traceability of results. Power grid operators can directly access the model, but the internal structure of the data-driven black-box model is invisible. Moreover, DBRB-I can identify key parameters of the PV power generation system, and experts can further improve their expert knowledge by analyzing the key parameters. The sensitivity analysis for each rule of Sub-BRB3 is shown in Fig. 27. The parts drawn with black circles clearly show that there are jumps in the system, which can have a huge impact on the PV system when the rule weights are within a certain interval. Therefore, to ensure that the system can maintain a balance between accuracy and interpretability, interpretability constraint 2 is necessary. For the higher sensitivity rules 1, 2, 4, 5, and 7, grid operators should analyze the hidden mechanisms in detail as feedback on the actual situation^38^. For the less sensitive rules 3, 6, 8, and 9, the modelling process should be further improved, which can improve the modelling accuracy^38^.

**Figure 28:**
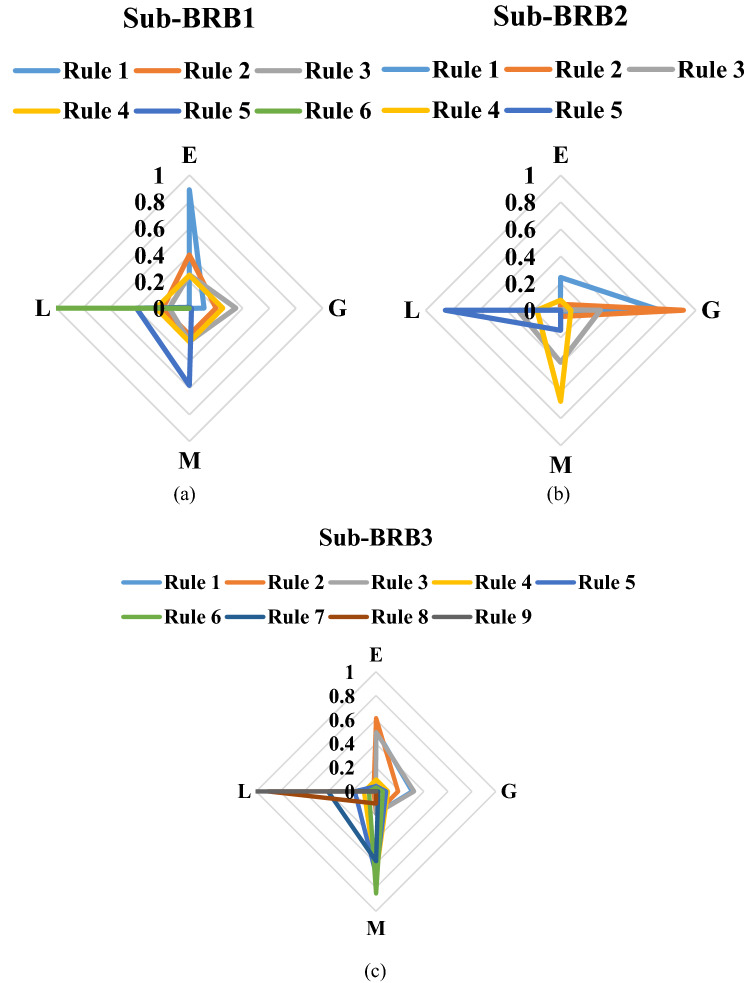
Belief distribution of each rule of DBRB-I(20).

The reasoning process and modelling process of the DBRB-I(20) model can be accessed by decision makers and users. Therefore, DBRB-I as an interpretability model can effectively analyse the system. Each rule of Sub-BRB1, Sub-BRB2, and Sub-BRB3 of DBRB-I(20) is shown in Fig. 28. As shown in Figure a, Sub-BRB1 has low sensitivity to the belief level G but high sensitivity to E and L. The reason for this is that the irradiance, voltage and output power data of the model are not well fitted at the belief level G, as shown in Fig. 6, which is consistent with reality. As shown in Figure b, Sub-BRB2 has low sensitivity to belief level E but high sensitivity to G, L, and M. The reason for this is that the module temperatures of the system do not reach the belief level of E, as shown in Fig. 6. As shown in Figure c, Sub-BRB3 combines Sub-BRB1 and Sub-BRB2 and is sensitive to belief levels E, G, M, and L. This further demonstrates the effectiveness of the DBRB-I(20) model.

## Conclusion

In this paper, a new PV power generation prediction model based on deep belief rule base with interpretability (DBRB-I) is proposed. In the DBRB-I model, first, the attributes of the PV power generation system are trended toward analysis, and the Sub-BRB is constructed through the correlation between the attributes and the results. Second, sensitivity analysis of the initial DBRB-I model constructed by experts, which reduces inefficient rules and redundant rules to reduce model complexity. Finally, the simplified DBRB-I model is optimized by an interpretability optimization algorithm.

There are three innovations in this paper. A PV power generation prediction model is proposed based on the DBRB-I model. The DBRB-I model consists of multiple Sub-BRBs in a deep structure, which effectively solves the problem of rule explosion and weak scalability of BRBs. To ensure that the interpretability of the model after optimization is not destroyed, a new optimization method is designed. Moreover, to improve the readability of PV power generation prediction models, a transparent and reliable rule reduction method sensitivity analysis is proposed. A case study of a PV power generation system is used to verify the validity of the proposed model. The results show that the DBRB-I model can maintain a good balance between interpretability and accuracy.

However, the local sensitivity analysis method used in this paper has limitations. This method neglects the mutual influence between uncertain parameters, which will interfere with the decision-making results. Therefore, how to use global sensitivity analysis deserves further study. Moreover, more benchmark datasets should be used and how to adequately analyze and interpret the sensitivity of a model in future research.

## Supplementary Information

Supplementary Information.

## Data Availability

The datasets generated and analysed during the current study are available in the AI Studio repository, https://aistudio.baidu.com/aistudio/datasetdetail/147402.
