# Supplementary material for: Deep belief rule based photovoltaic power forecasting method with interpretability
Source: Sci Rep. 2022 Aug 24;12:14467. doi: 10.1038/s41598-022-18820-6 (PMC9402627; doi:10.1038/s41598-022-18820-6)
Supplement: Supplementary file 1 — Supplementary Information. [file 41598_2022_18820_MOESM1_ESM.docx]

**Appendix Table A**

Table A1 Initial belief and constraints for Sub-BRB1 in DBRB-I.

| NO. | Attribute | | The initial belief | The belief constraint |
| --- | --- | --- | --- | --- |
|  | Irriadiance | Voltage | $\left\{\beta_{1},\beta_{2},\beta_{3},\beta_{4}\right\}$ | $\left\{\beta_{1},\beta_{2},\beta_{3},\beta_{4}\right\}$ |
| 1 | E | E | {0.95, 0.05, 0.00, 0.00} | {0.80 ~ 0.95, 0.10 ~ 0.20, 0.00 ~ 0.05, 0.00 ~ 0.05} |
| 2 | E | G | {0.72, 0.23, 0.05, 0.00} | {0.40 ~ 0.80, 0.20 ~ 0.40, 0.00 ~ 0.20, 0.00 ~ 0.20} |
| 3 | E | M | {0.62, 0.29, 0.09, 0.00} | {0.40 ~ 0.80, 0.10 ~ 0.50, 0.00 ~ 0.30, 0.00 ~ 0.20} |
| 4 | E | L | {0.48, 0.04, 0.12, 0.00} | {0.30 ~ 0.70, 0.40 ~ 0.60, 0.00 ~ 0.40, 0.00 ~ 0.20} |
| 5 | G | E | {0.52, 0.40, 0.08, 0.00} | {0.30 ~ 0.70, 0.20 ~ 0.65, 0.00 ~ 0.30, 0.00 ~ 0.10} |
| 6 | G | G | {0.47, 0.48, 0.15,0.00} | {0.25 ~ 0.50, 0.30 ~ 0.50, 0.00 ~ 0.25, 0.00 ~ 0.15} |
| 7 | G | M | {0.42, 0.41, 0.17,0.00} | {0.20 ~ 0.65, 0.20 ~ 0.60, 0.00 ~ 0.30, 0.00 ~ 0.10} |
| 8 | G | L | {0.40, 0.40, 0.20, 0.00} | {0.20 ~ 0.60, 0.20 ~ 0.60, 0.00 ~ 0.40, 0.00~ 0.20} |
| 9 | M | E | {0.24, 0.32, 0.44, 0.00} | {0.10 ~ 0.50, 0.20 ~ 0.60, 0.20 ~ 0.75, 0.00 ~ 0.10} |
| 10 | M | G | {0.15, 0.16, 0.46, 0.03} | {0.10 ~ 0.25, 0.10 ~ 0.25, 0.25 ~ 0.60, 0.10 ~ 0.35} |
| 11 | M | M | {0.00, 0.17, 0.51, 0.32} | {0.00 ~ 0.10, 0.00 ~ 0.20, 0.40 ~ 0.75, 0.30 ~ 0.40} |
| 12 | M | L | {0.00, 0.12, 0.46, 0.42} | {0.00 ~ 0.15, 0.00 ~ 0.35, 0.20 ~ 0.70, 0.20 ~ 0.70} |
| 13 | L | E | {0.05, 0.25, 0.23, 0.47} | {0.00 ~ 0.25, 0.10 ~ 0.50, 0.10 ~ 0.50, 0.20 ~ 0.75} |
| 14 | L | G | {0.00, 0.14, 0.36, 0.50} | {0.00 ~ 0.20, 0.00 ~ 0.35, 0.10 ~ 0.70, 0.30 ~ 0.80} |
| 15 | L | M | {0.00, 0.05, 0.10, 0.85} | {0.00 ~ 0.10, 0.00 ~ 0.10, 0.00 ~ 0.20, 0.80 ~ 1.00} |
| 16 | L | L | {0.00, 0.00, 0.00, 1.00} | {0.00 ~ 0.10, 0.00 ~ 0.10, 0.00 ~ 0.20, 0.85 ~ 1.00} |

Table A2 Initial belief and constraints for Sub-BRB2 in DBRB-I.

| NO. | Attribute | | The initial belief | The belief constraint |
| --- | --- | --- | --- | --- |
|  | y1 | Module temperature | $\left\{\beta_{1},\beta_{2},\beta_{3},\beta_{4}\right\}$ | $\left\{\beta_{1},\beta_{2},\beta_{3},\beta_{4}\right\}$ |
| 1 | E | E | {0.90, 0.10, 0.00, 0.00} | {0.60 ~ 1.00, 0.00 ~ 0.40, 0.00 ~ 0.10, 0.00 ~ 0.10} |
| 2 | E | G | {0.70, 0.20, 0.10, 0.00} | {0.45 ~ 1.00, 0.00 ~ 0.50, 0.00 ~ 0.30, 0.00 ~ 0.10} |
| 3 | E | M | {0.65, 0.20, 0.15, 0.00} | {0.45 ~ 0.85, 0.00 ~ 0.50, 0.00 ~ 0.40, 0.00 ~ 0.10} |
| 4 | E | L | {0.40, 0.40, 0.20, 0.00} | {0.10 ~ 0.60, 0.10 ~ 0.60, 0.00 ~ 0.45, 0.00 ~ 0.10} |
| 5 | G | E | {0.15, 0.80, 0.05, 0.00} | {0.00 ~ 0.40, 0.60 ~ 1.00, 0.00 ~ 0.20, 0.00 ~ 0.00} |
| 6 | G | G | {0.15, 0.75, 0.10,0.00} | {0.10 ~ 0.45, 0.40 ~ 0.80, 0.00 ~ 0.20, 0.00 ~ 0.05} |
| 7 | G | M | {0.20, 0.61, 0.19,0.00} | {0.00 ~ 0.25, 0.50 ~ 0.95, 0.00 ~ 0.30, 0.00 ~ 0.10} |
| 8 | G | L | {0.30, 0.50, 0.15, 0.05} | {0.10 ~ 0.60, 0.20 ~ 0.70, 0.00 ~ 0.45, 0.00~ 0.15} |
| 9 | M | E | {0.20, 0.22, 0.42, 0.16} | {0.10 ~ 0.45, 0.10 ~ 0.50, 0.20 ~ 0.70, 0.00 ~ 0.45} |
| 10 | M | G | {0.05, 0.15, 0.60, 0.20} | {0.00 ~ 0.10, 0.10 ~ 0.40, 0.30 ~ 0.70, 0.15 ~ 0.40} |
| 11 | M | M | {0.00, 0.10, 0.75, 0.15} | {0.00 ~ 0.10, 0.00 ~ 0.15, 0.60 ~ 0.90, 0.10 ~ 0.25} |
| 12 | M | L | {0.00, 0.05, 0.80, 0.15} | {0.00 ~ 0.20, 0.00 ~ 0.25, 0.60 ~ 0.10, 0.00 ~ 0.30} |
| 13 | L | E | {0.00, 0.10, 0.15, 0.75} | {0.00 ~ 0.15, 0.00 ~ 0.25, 0.00 ~ 0.45, 0.50 ~ 0.90} |
| 14 | L | G | {0.00, 0.00, 0.22, 0.78} | {0.00 ~ 0.10, 0.00 ~ 0.10, 0.10 ~ 0.50, 0.60 ~ 1.00} |
| 15 | L | M | {0.00, 0.00, 0.20, 0.80} | {0.00 ~ 0.10, 0.00 ~ 0.15, 0.15 ~ 0.30, 0.75 ~ 1.00} |
| 16 | L | L | {0.00, 0.00, 0.10, 0.90} | {0.00 ~ 0.10, 0.00 ~ 0.10, 0.00 ~ 0.30, 0.80 ~ 1.00} |

Table A3 Initial belief and constraints for Sub-BRB3 in DBRB-I.

| NO. | Attribute | | The initial belief | The belief constraint |
| --- | --- | --- | --- | --- |
|  | y2 | Ambient temperature | $\left\{\beta_{1},\beta_{2},\beta_{3},\beta_{4}\right\}$ | $\left\{\beta_{1},\beta_{2},\beta_{3},\beta_{4}\right\}$ |
| 1 | E | E | {1.00, 0.00, 0.00, 0.00} | {0.90 ~ 1.00, 0.00 ~ 0.10, 0.00 ~ 0.10, 0.00 ~ 0.10} |
| 2 | E | G | {0.73, 0.27, 0.00, 0.00} | {0.50 ~ 0.90, 0.10 ~ 0.50, 0.00 ~ 0.10, 0.00 ~ 0.10} |
| 3 | E | M | {0.61, 0.30, 0.09, 0.00} | {0.40 ~ 0.85, 0.10 ~ 0.60, 0.00 ~ 0.20, 0.00 ~ 0.10} |
| 4 | E | L | {0.54, 0.24, 0.22, 0.00} | {0.20 ~ 0.80, 0.10 ~ 0.55, 0.10 ~ 0.50, 0.00 ~ 0.10} |
| 5 | G | E | {0.60, 0.40, 0.00, 0.00} | {0.30 ~ 0.70, 0.30 ~ 0.40, 0.00 ~ 0.20, 0.00 ~ 0.15} |
| 6 | G | G | {0.65, 0.20, 0.15,0.00} | {0.60 ~ 0.75, 0.15 ~ 0.25, 0.00 ~ 0.20, 0.00 ~ 0.10} |
| 7 | G | M | {0.40, 0.40, 0.20,0.00} | {0.40 ~ 0.70, 0.10 ~ 0.40, 0.10 ~ 0.20, 0.00 ~ 0.15} |
| 8 | G | L | {0.30, 0.50, 0.20, 0.00} | {0.10 ~ 0.55, 0.30 ~ 0.80, 0.10 ~ 0.50, 0.00~ 0.10} |
| 9 | M | E | {0.14, 0.28, 0.53, 0.05} | {0.00 ~ 0.15, 0.10 ~ 0.30, 0.50 ~ 0.70, 0.00 ~ 0.10} |
| 10 | M | G | {0.05, 0.06, 0.66, 0.23} | {0.00 ~ 0.10, 0.00 ~ 0.10, 0.60 ~ 0.90, 0.10 ~ 0.30} |
| 11 | M | M | {0.00, 0.10, 0.68, 0.22} | {0.00 ~ 0.10, 0.00 ~ 0.15, 0.60 ~ 0.90, 0.00 ~ 0.25} |
| 12 | M | L | {0.00, 0.05, 0.68, 0.27} | {0.00 ~ 0.05, 0.00 ~ 0.20, 0.40 ~ 0.70, 0.10 ~ 0.40} |
| 13 | L | E | {0.00, 0.01, 0.25, 0.74} | {0.00 ~ 0.10, 0.00 ~ 0.10, 0.00 ~ 0.50, 0.50 ~ 0.90} |
| 14 | L | G | {0.00, 0.00, 0.20, 0.80} | {0.00 ~ 0.10, 0.00 ~ 0.10, 0.10 ~ 0.25, 0.80 ~ 1.00} |
| 15 | L | M | {0.00, 0.01, 0.20, 0.97} | {0.00 ~ 0.05, 0.00 ~ 0.05, 0.00 ~ 0.15, 0.90 ~ 1.00} |
| 16 | L | L | {0.00, 0.00, 0.00, 1.00} | {0.00 ~ 0.10, 0.00 ~ 0.10, 0.00 ~ 0.10, 0.90 ~ 1.00} |

Table A4 Initial rule weights and constraints for Sub-BRBn in DBRB-I

| NO. | The initial rule weights | The rule weights constraint |
| --- | --- | --- |
| 1 | 1 | 0.4 ~ 1 |
| 2 | 1 | 0.4 ~ 1 |
| 3 | 1 | 0.4 ~ 1 |
| 4 | 1 | 0.4 ~ 1 |
| 5 | 1 | 0.4 ~ 1 |
| 6 | 1 | 0.4 ~ 1 |
| 7 | 1 | 0.4 ~ 1 |
| 8 | 1 | 0.4 ~ 1 |
| 9 | 1 | 0.4 ~ 1 |
| 10 | 1 | 0.4 ~ 1 |
| 11 | 1 | 0.4 ~ 1 |
| 12 | 1 | 0.4 ~ 1 |
| 13 | 1 | 0.4 ~ 1 |
| 14 | 1 | 0.4 ~ 1 |
| 15 | 1 | 0.4 ~ 1 |
| 16 | 1 | 0.4 ~ 1 |

Table A5 Initial attribute weights and constraints in DBRB-I

| Attribute | Attribute weight | Attribute weights constraint |
| --- | --- | --- |
| Irriadiance | 1 | 0.6-1 |
| Voltage | 1 | 0.6-1 |
| Module temperature | 1 | 0.6-1 |
| Ambient temperature | 1 | 0.4-1 |

**Appendix Table B**

Table B1 Optimized belief for Sub-BRB1 in DBRB-I

| NO. | Rule weight | Attribute weight | | The Optimized belief |
| --- | --- | --- | --- | --- |
|  |  | Irriadiance | Voltage | $\left\{\beta_{1},\beta_{2},\beta_{3},\beta_{4}\right\}$ |
|  |  | 0.60 | 0.60 |  |
| 1 | 1.00 | E | E | {0.89, 0.11, 0.00, 0.00} |
| 2 | 1.00 | E | G | {0.40, 0.20, 0.20, 0.20} |
| 3 | 0.60 | G | G | {0.25, 0.35, 0.25,0.15} |
| 4 | 0.83 | M | G | {0.25, 0.25, 0.25, 0.25} |
| 5 | 0.98 | M | M | {0.01, 0.02, 0.58, 0.40} |
| 6 | 0.60 | L | M | {0.00, 0.00, 0.00, 1.00} |

Table B2 Optimized belief for Sub-BRB2 in DBRB-I

| NO. | Rule weight | Attribute weight | | The Optimized belief |
| --- | --- | --- | --- | --- |
|  |  | y1 | Module temperature | $\left\{\beta_{1},\beta_{2},\beta_{3},\beta_{4}\right\}$ |
|  |  | 0.62 | 0.99 |  |
| 1 | 0.60 | G | G | {0.24, 0.74, 0.01,0.00} |
| 2 | 0.80 | G | M | {0.04, 0.91, 0.05,0.00} |
| 3 | 0.79 | M | G | {0.00, 0.29, 0.38, 0.32} |
| 4 | 0.80 | M | M | {0.07, 0.08, 0.67, 0.17} |
| 5 | 1.00 | L | M | {0.00, 0.00, 0.15, 0.85} |

Table B3 Optimized belief for Sub-BRB3 in DBRB-I

| NO. | Rule weight | Attribute weight | | The Optimized belief |
| --- | --- | --- | --- | --- |
|  |  | y2 | Ambient temperature | $\left\{\beta_{1},\beta_{2},\beta_{3},\beta_{4}\right\}$ |
|  |  | 0.63 | 0.40 |  |
| 1 | 0.40 | G | E | {0.51, 0.30, 0.19, 0.00} |
| 2 | 0.60 | G | G | {0.60, 0.20, 0.20,0.00} |
| 3 | 1.00 | G | M | {0.52, 0.27, 0.21,0.00} |
| 4 | 0.43 | M | E | {0.00, 0.10, 0.82, 0.08} |
| 5 | 0.70 | M | G | {0.04, 0.07, 0.83, 0.06} |
| 6 | 1.00 | M | M | {0.03, 0.06, 0.85, 0.06} |
| 7 | 0.400 | M | L | {0.00, 0.00, 0.60, 0.40} |
| 8 | 0.87 | L | G | {0.00, 0.00, 0.10, 0.90} |
| 9 | 1.00 | L | M | {0.00, 0.00, 0.00, 1.00} |

Table B4 Execution time of different models

| Model | Number of rule | Execution time |
| --- | --- | --- |
| DBRB-I(20) | 20 | 80.34s |
| DBRB(41) | 41 | 60.13s |
| DBRB(48) | 48 | 61.02s |
